# Itaconate Suppresses the Activation of Mitochondrial NLRP3 Inflammasome and Oxidative Stress in Allergic Airway Inflammation

**DOI:** 10.3390/antiox12020489

**Published:** 2023-02-15

**Authors:** Qiu-Meng Xie, Ning Chen, Si-Ming Song, Cui-Cui Zhao, Ya Ruan, Jia-Feng Sha, Qian Liu, Xu-Qin Jiang, Guang-He Fei, Hui-Mei Wu

**Affiliations:** 1 Anhui Geriatric Institute, Department of Geriatric Respiratory and Critical Care Medicine, The First Affiliated Hospital of Anhui Medical University, Jixi Road 218, Hefei 230022, China; 2 Key Laboratory of Geriatric Molecular Medicine of Anhui Province, Jixi Road 218, Hefei 230022, China; 3 Key Laboratory of Respiratory Disease Research and Medical Transformation of Anhui Province, Jixi Road 218, Hefei 230022, China; 4 Division of Life Sciences and Medicine, University of Science and Technology of China, Hefei 230026, China; 5 Department of Respiratory Medicine, The First Affiliated Hospital of University of Science and Technology of China, Hefei 230001, China

**Keywords:** allergic airway inflammation, itaconate, Irg1, alveolar macrophage, mitochondrial, NLRP3 inflammasome, oxidative stress

## Abstract

Itaconate has emerged as a novel anti-inflammatory and antioxidative endogenous metabolite, yet its role in allergic airway inflammation (AAI) and the underlying mechanism remains elusive. Here, the itaconate level in the lung was assessed by High Performance Liquid Chromatography (HPLC), and the effects of the Irg1/itaconate pathway on AAI and alveolar macrophage (AM) immune responses were evaluated using an ovalbumin (OVA)-induced AAI model established by wild type (WT) and *Irg1*^−/−^ mice, while the mechanism of this process was investigated by metabolomics analysis, mitochondrial/cytosolic protein fractionation and transmission electron microscopy in the lung tissues. The results demonstrated that the *Irg1* mRNA/protein expression and itaconate production in the lung were significantly induced by OVA. Itaconate ameliorated while *Irg1* deficiency augmented AAI, and this may be attributed to the fact that itaconate suppressed mitochondrial events such as NLRP3 inflammasome activation, oxidative stress and metabolic dysfunction. Furthermore, we identified that the Irg1/itaconate pathway impacted the NLRP3 inflammasome activation and oxidative stress in AMs. Collectively, our findings provide evidence for the first time, supporting the conclusion that in the allergic lung, the itaconate level is markedly increased, which directly regulates AMs’ immune responses. We therefore propose that the Irg1/itaconate pathway in AMs is a potential anti-inflammatory and anti-oxidative therapeutic target for AAI.

## 1. Introduction

Asthma is a common chronic airway inflammatory disease, with more than 300 million people suffering worldwide [1]. It is characterized by reversible airflow obstruction, bronchial hyperresponsiveness and airway inflammation [1]. Although asthma can be largely controlled, about 5–10% of asthmatic patients are poorly controlled by the conventional therapy and strategy such as inhaled corticosteroids [2]. Moreover, the side effects caused by the excessive use of inhaled corticosteroids (ICS) result in the increasingly high burden of this disease [3].

Itaconate is generated by the decarboxylation of tricarboxylic acid (TCA) cycle intermediate *cis*-aconitate by aconitate decarboxylase which is encoded by the immune response gene 1 (*Irg1*) [4]. It is increasingly considered as an important small immune metabolite with significant anti-inflammatory and anti-oxidative activities [5]. Endogenous itaconate is evidenced to be upregulated [6,7,8,9] or downregulated [10,11] in several inflammatory diseases. Exogenous administration of itaconate derivatives reportedly inhibits airway inflammation caused by infection. For example, tetraoctyl itaconate (4-OI) decreases the replication of respiratory viruses such as SARS-CoV2, Zika and influenza, accompanied with reduced airway inflammation and pathological changes caused by these respiratory viruses [12]. 4-OI has been also demonstrated to attenuate the manifestations of airway inflammation caused by allergens [13,14]. However, in contrast to itaconate derivatives such as 4-OI, knowledge on the role of non-derivatized itaconate in allergic airway inflammation (AAI) is still limited, and the underlying mechanism remains incompletely understood.

As the key enzyme for itaconate synthesis, *Irg1* is mainly expressed in activated immune cells, and is up-regulated nearly 200-fold in activated macrophages [15]. As we know, alveolar macrophages (AMs) account for more than 90% of bronchoalveolar lavage fluid cells under homeostatic conditions. Together with airway epithelial cells, they constitute the first line of defense in the respiratory tract, and their role in the pathogenesis of allergen-induced asthma has attracted more and more attention [16]. Macrophage activation is accompanied by tightly controlled intracellular metabolic reprogramming, in which itaconate is a hallmark. Several studies have implicated the significant role of 4-OI in regulating bone marrow-derived macrophage (BMDM) immune responses upon LPS stimulation such as inhibiting the production of pro-inflammatory factors, NOD-like receptor protein 3 (NLRP3) inflammasome activation and M2 polarization [5,14,17,18]. However, the effect of itaconate on AMs’ inflammatory response in AAI remains largely unexplored.

The inflammatory response and oxidative stress are closely related and reinforce each other [19]. Mitochondria are the powerhouse of the cell, and are also the major source of cellular ROS, lying at the heart of immunity [20]. Such mitochondrial function is associated with the according changes in their morphology and ultrastructure, as mitochondrial fission mediated by the dynamin-related protein 1 (DRP1) and the mitochondrial fission 1 protein (Fis1) triggers fragmentation, while the fusion mediated by Optic atrophy (OPA1) and mitofusions (Mfn1 and Mfn2) drives mitochondria elongation, and the mitochondrial dynamic balance protects the cell against oxidative stress [21]. Moreover, mitochondria are also critical for the activation of the NLRP3 inflammasome. Studies have implicated that the translocation of NLRP3 from the endoplasmic reticulum (ER) to mitochondria or mitochondria-associated ER membranes is considered to precede NLRP3 inflammasome assembly and activation [22,23]. Moreover, a link between mitochondrial malfunction, ROS and NLRP3 inflammasome activation has been proposed in allergic airway diseases [24] and chronic obstructive pulmonary disease [25]. Considering the effective anti-inflammatory and anti-oxidative function of itaconate, we speculated that itaconate may represent an intrinsic negative regulator of AMs’ inflammatory response in allergic airway diseases, where it regulates the NLRP3 inflammasome activation and oxidative stress.

Therefore, in the present study, we firstly investigated whether the level of endogenous itaconate was changed in a murine AAI model, then studied the effect of the exogenous administration of itaconate or deletion of *Irg1* on airway inflammation and on primary AMs’ immune responses in response to the repeated OVA challenge, and revealed the underlying regulatory mechanism of itaconate in AAI.

## 2. Materials and Methods

### 2.1. Mice

Female C57BL/6 mice (6 weeks, 16–18 g) were obtained from GemPharmatech Co., Ltd. (Nan Jing, China). The *Irg1*^−/−^ mice were available from Cyagen Biosciences Inc. (gene ID: 16365, Stock# S-KO-02680, Suzhou, China). *Irg1*^−/−^ mice were viable, fertile and kept in specific pathogen-free conditions for reproduction. Homozygous *Irg1*^−/−^ mice were generated by intercrossing the heterozygous mice and confirmed by Polymerase Chain Reaction (PCR) (F1: 5′-TGTTACAGTCAGAGATGGAGAGG-3′, R1: 5′-CACACACAGCAGCTCACAGTAG-3′; and F1: 5′-TGTTACAGTCAGAGATGGAGAGG-3′, R2: 5′-TGTGTCA GGTACGGTAATGAGTG-3′). All animal experiments were in accordance with the Committee on the Ethics of Animal Care and Use of Anhui Medical University (LLSC20210909).

### 2.2. Murine AAI Model

C57BL/6 and *Irg1*^−/−^ mice were sensitized by two intraperitoneal injections of 10 µg OVA (Sigma, St. Louis, MO, USA) complexed with 1 mg potassium aluminum sulfate (Sangon Biotech, Shanghai, China) or saline on day 0 and day 7, then challenged with 1% aerosolized OVA or saline for 30 min per day on days 14–20. Additionally, mice received intraperitoneal injections of itaconate (30 mg/kg, 100 mg/kg, 150 mg/kg, Sigma) or MitoTEMPO (5 mg/kg, Sigma) before each OVA challenge. All mice were sacrificed on day 21; after collecting serum, the left lung was subjected to a bronchoalveolar lavage and subsequent differential cell counting and ELISA analysis, while the right lung of each mouse was used for a histopathological analysis, immunohistochemistry and further Western blotting assays.

### 2.3. Bronchoalveolar Lavage Fluid (BALF) Collection and Lung Histopathology

The mice were completely anesthetized with 1% sodium pentobarbital (100 mg/kg); then, bronchoalveolar lavage (BAL) was performed thrice by lavaging the left lung with 0.5 mL of ice-cold PBS. The collected BALF were centrifuged at 700× *g* at 4 °C for 5 min, and the cell pellets harvested from BALF were re-suspended in 200 µL Phosphate-buffered saline (PBS). The total cells were counted using a hemocytometer, while differential cell counts were determined by staining cytospins of BALF samples with Wright–Giemsa (Baso, Zhuhai, China). At least 200 cells were counted for each mouse. The right lungs were fixed in 4% paraformaldehyde, dehydrated through gradient ethanol and embedded in paraffin. Then, the paraffin-embedded lungs were cut into 5 μm and stained with hematoxylin/eosin (H&E) or periodic acid–Schiff (PAS) to analyze the inflammation and mucus productions, respectively.

### 2.4. Enzyme-Linked Immunosorbent Assay (ELISA)

The levels of OVA-specific IgE in the serum were measured by ELISA using a specific kit from Cusabio (Wuhan, China); the levels of IL-5, IL-4, IL-13, IL-1β and IL-18 in BALF were measured by ELISA kits from Cloud-Clone Crop (Wuhan, China), according to the protocols from the manufacturer.

### 2.5. Tricarboxylic Acid (TCA) Cycle Organic Acid Metabolomics Analysis

Fresh lung tissue was washed with ice-cold PBS and immediately frozen in liquid nitrogen. Three samples per group were collected and sent for a TCA organic acid (OA) metabolomics’ analysis (LipidALL Technologies, Changzhou, China). The organic acid involved in the TCA cycle were extracted from murine lung tissue using acetonitrile: water (1:1) and derivatized using 3-nitrophenylhdyrazone, then were analyzed on a Jasper HPLC coupled to a Sciex 4500 MD system. In brief, individual organic acids were separated on a Phenomenex Kinetex C18 column (100 × 2.1 mm, 2.6 μm) using 0.1% formic acid (Sigma) in water as the mobile phase A and 0.1% formic acid in acetonitrile (Sigma) as the mobile phase B. d^4^-succinic acid, d^4^-citric acid, d^3^-malic acid, ^13^C-3-lactate acid, d^3^-pyruvate acid and d^4^-fumarate acid purchased from Cambridge Isotope Laboratories (Tewksbury, MA, USA) were used as internal standards for the quantitation.

### 2.6. Energy Metabolomics Analysis

After washing with ice-cold PBS, fresh lung tissues were immediately frozen in liquid nitrogen. Three samples per group were collected and sent for energy metabolomics analysis (Applied Protein Technology, Shanghai, China). A homogenate of 100 mg of the sample mixed with 1 mL of cold methanol/acetonitrile/H_2_O (2:2:1, *v*/*v*/*v*) was sonicated at a low temperature (30 min/once, twice) and then centrifuged for 20 min (14,000× *g*, 4 °C). The supernatant was dried in a vacuum centrifuge. For the LC-MS analysis, the dried samples were dissolved in 100 μL of acetonitrile/water (1:1, *v*/*v*), adequately vortexed and then centrifuged (14,000 rpm, 4 °C, 15 min). The supernatants were collected for the LC-MS/MS analysis. Analyses were performed using a UHPLC (1290 Infinity LC, Agilent Technologies, Santa Clara, California, USA) coupled with a QTRAP (AB Sciex 5500, Framingham, CT, USA).

### 2.7. Lung Tissue Mitochondrial and Cytosolic Protein Fractionation

The isolation of lung tissue mitochondria and cytosol was performed using the Tissue Mitochondria Isolation Kit (Beyotime, Shanghai, China). Briefly, 100 mg of lung tissue was cut into small pieces, then the Mitochondrial Isolation Solution containing phenylmethanesulfonyl fluoride (PMSF) was added, and the mixture was homogenated using a tissuelyzer (JINGXIN, Shanghai, China). The homogenate was centrifuged at 1000× *g* for 5 min at 4 °C; then, the supernatant was collected and centrifuged at 11,000× *g* for 10 min at 4 °C to isolate the mitochondria (deposition) fractions. The supernatant was further centrifuged at 12,000× *g* for 10 min to obtain cytosolic proteins. Samples of mitochondria were dissolved in the Mitochondrial Lysis Solution to obtain mitochondrial proteins. COX IV (Abcam, Boston, MA, USA) was used to verify the efficiency of isolation by immunoblot.

### 2.8. Lung Tissue Transmission Electron Microscopy (TEM)

The lung tissue (1 mm × 1 mm × 1 mm) was fixed at 4 °C overnight in freshly prepared 2.5% glutaraldehyde fixative, followed by washing in PBS for about 6 h. Then, tissues were soaked in 1% osmium tetroxide for 2 h at 4 °C and dehydrated in sequential 30% and 70% ethanol, followed by staining with 70% uranium acetate and dehydrated in gradient ethanol, propylene oxide, propylene oxide and epoxy resin (1:1). The tissues were embedded in epoxy resin, and 70 nm sections were cut by ul-tramicrotome (Leica EM, UC7, Wetzlar, Germany) and were post-stained in uranyl acetate and bismuth subnitrate. The sections were observed under TEM (JEM1400, JEOL, Tokyo Metropolis, Japan), and the images were recorded on morada G3 of EMSIS GMBH, Münster, Germany.

### 2.9. Immunohistochemistry (IHC)

The immunohistochemistry was performed using Biotin-Streptavidin HRP Detection Systems (ZSGB-BIO, Beijing, China). The embedded lung tissue was cut into 5 μm sections, followed by dewaxing, hydrating and washing in PBS. Then, endogenous peroxidase was blocked and antigen repair was performed. Sections were incubated with corresponding primary antibodies at 37 °C for 1 h and then overnight at 4 °C with antibodies binding to 8-OHdG, nitrotyrosine, SOD2, OPA1, Mfn2, DRP1, Fis1 and F4/80 (all from Santa Cruz, Dallas, TX, USA); F4/80 staining was used to identify the macrophage. The next day, the sections were incubated with the biotin-labeled goat anti-mouse/rabbit IgG and horseradish peroxidase streptavidin. Finally, the visualization was accomplished with freshly prepared diaminobenzidine (DAB), and the resulting judgement was carried out under an optical microscope (Leica, Germany).

### 2.10. Detection of Reactive Oxygen Species (ROS) in the Lung

The fluorescent probe dihydroethidium (DHE, Sigma) was used to detect the ROS level in the lung according to the previous report with a minor modification [26]: DHE can enter cells and is oxidized by a superoxide to emit red fluorescence. When systemically administered to animals, DHE can rapidly distribute into various tissues [27]. Four hours before sacrifice, 10 mg/kg DHE was injected into mice through the tail vein. Then, the lung tissue was quickly collected, and frozen sections were made by sucrose dehydration and OCT embedding. A 4,6-diamidino-2-phenylindole (DAPI) solution was used to stain the nuclei. The lung slices under a 535 nm excitation wavelength were observed by a laser confocal microscope (ZEISS LSM880, Jena, Germany).

### 2.11. Primary Alveolar Macrophage (AM) Isolation and Culture

AMs were harvested from the BALF of mice, as previously described [28]. Briefly, the lungs of the WT and *Irg1*^−/−^ mice were lavaged with 0.8 mL of cold phosphate-buffered saline (PBS) for four times to collect BALF. Each BALF sample was centrifuged at 700× *g* for 5 min, and the supernatant was discarded. The pellet (three or five mice) was pooled together and re-suspended in DMEM medium (Hyclone, Logan, UT, USA) to obtain a cell concentration of 2.5 × 10^5^ cells/well. After 30 min of incubation at 37 °C with 5% CO_2_, the unattached cells were removed, and the adherent cells were cultured in DMEM containing 10% fetal bovine serum (FBS; GBICO, Invitrogen, Waltham, MA, USA), 100 IU/mL penicillin and 100 µg/mL streptomycin. The purity of the AMs was >98% according to staining with Wright–Giemsa (Baso, Zhuhai, China) and flow cytometry for CD45^+^F4/80^+^CD11b^−^CD11c^+^ cells (BD Biosciences, San Jose, CA, USA). Then, AMs from WT and *Irg1*^−/−^ mice were incubated in the presence or absence of OVA (Sigma, 500 μg/mL) for 24 h to detect the protein expression of Irg1. 

On the other hand, BALF from the mice of the AAI model was collected as described above. After 30 min of incubation at 37 °C with 5% CO_2_, the unattached cells were removed and the attached cells were washed by PBS three times. Then, the AMs were immediately lysed by a RIPA reagent containing a protease inhibitor cocktail (Roche, Indianapolis, IN, USA) on ice for 20 min. 

### 2.12. Double-Labeling Immunofluorescence (IF)

AMs from the BALF of the vehicle or OVA-challenged mice were isolated as described above and seeded in 24-well plates containing a coverslip. After adherence, the cells were washed by PBS and fixed with 10% formaldehyde. After permeabilizing in PBS containing 0.5% Triton X-100 and blocked with 5% donkey serum, the cells were incubated with F4/80 (Santa Cruz) and Irg1 (Abcam, Boston, MA, USA) antibodies, and F4/80 staining was used to identify the macrophage. The second antibody was the Alexa Fluor 594^®^-conjugated anti rabbit or Alexa Fluor 488^®^-conjugated anti mouse IgG (Jackson ImmunoResearch Lab, Inc. West Grove, PA, USA). Then, the coverslips were counterstained with DAPI and observed with confocal microscopy (Zeiss LSM 800, Carl Zeiss AG, Oberkochen, Germany, 63 × 1.4 oil).

### 2.13. Immunoblot

Proteins were extracted from lung tissues and BALF AMs using a RIPA reagent containing a protease inhibitor cocktail (Roche, Indianapolis, IN, USA). Equal amounts of proteins were separated on 12% SDS-PAGE gels and transferred to PVDF membranes (Millipore, Billerica, MA, USA). Membranes were incubated with 5% non-fat milk for 1 h and then with primary antibodies overnight at 4 °C. The following primary antibodies were used: Irg1 and COX IV were obtained from Abcam (Boston, MA, USA), NLRP3 and Caspase 1(p20) were from Adipogen (San Diego, CA, USA), IL-1β (R&D Systems, Minneapolis, MN, USA), Nitrotyrosine, SOD2, OPA1, Mfn2, DRP1, Fis1, IL-4, IL-5 and IL-13 were all from Santa Cruz (CA, USA) and GAPDH (KANGCHEN Biotech, Shanghai, China). For the quantitative analysis, the intensity of the protein bands was determined by using Image J 1.38x software (NIH, Bethesda, MD, USA).

### 2.14. Quantitative PCR

The lung tissues were homogenized and the total RNA was isolated by using Trizol Reagent (Invitrogen, USA) and quantified using a one drop OD1000 spectrophotometer. cDNA was prepared by a reverse transcription PCR (RT-PCR). A quantitative PCR (qPCR) was performed by using SYBR Green Kit (Takara) and a StepOne Real Time PCR System (Applied Biosystems, Foster City, CA, USA). The primer pair sequences were as follows: *Irg1* (gene ID: 16365), FW: 5′-GCAACATGATGCTCAAGTCTG-3′ and RV: 5′- TGCTCCTCCGAATGATACCA-3′; β-actin (gene ID: 11461), FW: 5′- TGTTACCAACTGG GACGACA-3′ and RV: 5′ GGGGTGTTG AAGGTCTCAAA-3′. The 2^−ΔΔCt^ method was used to calculate the fold changes. 

### 2.15. Statistical Analysis

A statistical analysis was performed using GraphPad Prism 7.0 (GraphPad Software, Inc., San Diego, CA, USA). Data were expressed as mean ± SEM. A statistical comparison of the two groups was performed using the unpaired *t*-test. The differences among multiple groups were analyzed by using one-way ANOVA with post hoc Tukey tests. The threshold of statistical significance was designated to 0.05.

## 3. Results

### 3.1. Itaconate Level and Irg1 Expression Are Elevated in OVA-Induced AAI Model

**Figure 1:**
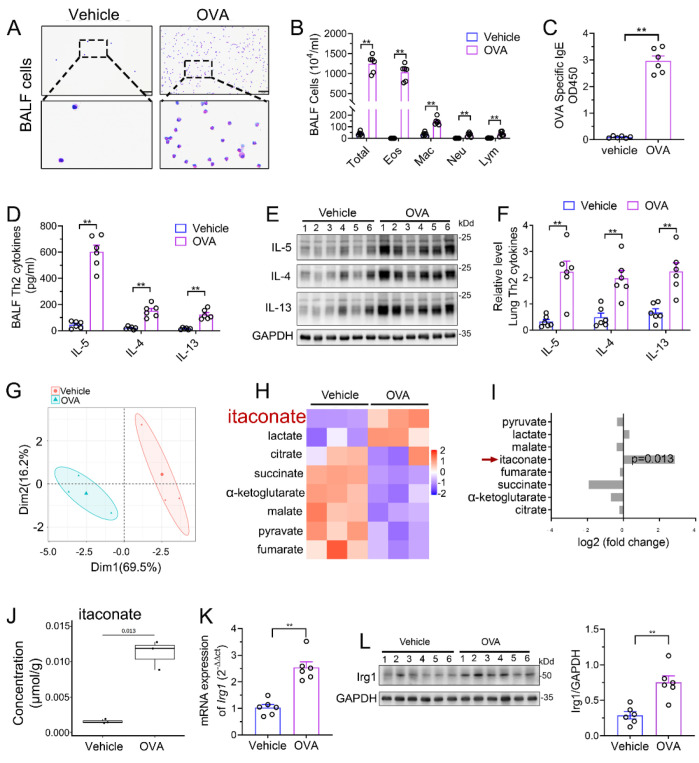
Itaconate level and *Irg1* expression are induced in OVA−induced allergic airway inflammation. (**A**) Representative images of BALF cells stained with Wright–Giemsa solution. Scale bar: 50 μm. (**B**) The number of total inflammatory cells, eosinophils, macrophages, neutrophils and lymphocytes in BALF. (**C**) The optical density value (OD value) measured at 450 nm represents the level of IgE in serum. (**D**) The level of Th2 cytokines IL-5, IL-4 and IL-13 in BALF. (**E**) Representative blots showing the protein expressions of Th2 cytokines IL-5, IL-4 and IL-13 in lung tissues from vehicle and OVA groups. (**F**) Quantification of the expression of IL-5, IL-4 and IL-13 in the lung as referencing to GAPDH. (**G**–**J**) TCA organic acid metabolomics analysis in vehicle and OVA group. *n* = 3 per group. (**G**) Principal component analysis (PCA) in vehicle and OVA group. (**H**) Heatmap of metabolites obtained by TCA organic acid metabolomics analysis in vehicle and OVA group. (**I**) Fold change of metabolites in OVA group as compared with vehicle group. (**J**) The level of itaconate in lung tissue. (**K**) The mRNA expression of *Irg1* in lung tissue. (**L**) Representative blots showing the protein expressions of Irg1 in lung tissues from vehicle and OVA groups and quantification. Cropped blots are shown, and Supplementary File S2: Figures S1 and S2 present the full-length blots. Data expressed as means ± SEM (*n* = 6). ** *p* < 0.01.

We established a murine AAI model by OVA sensitization and challenge. Airway inflammation including peribronchial inflammatory cells’ infiltration (Supplementary File S1: Figure S1), mucus secretion (Supplementary File S1: Figure S1), BALF inflammatory cell counts (Figure 1A,B), serum OVA-specific IgE level (Figure 1C) and Th2 cytokines’ production (Figure 1D–F) were significantly increased in the model group as compared to those in vehicle group, indicating the success of the model. Next, we performed an analysis of TCA cycle metabolites in the lung tissue to identify the changes of itaconate between the model group and vehicle group. The principal component analysis (PCA) demonstrated a significant separation between the vehicle and model group (Figure 1G), and the level of itaconate was elevated about sevenfold in the OVA-treated animals in comparison to the vehicle group (Figure 1H–J). Consistently, the mRNA and protein level of Irg1, which drives the itaconate production, were markedly increased in the lung of the OVA group (Figure 1K,L). These data collectively demonstrated that OVA sensitization and challenge induced the production of itaconate and its synthetase Irg1 expression in the lung.

### 3.2. Exogenous Itaconate Attenuates OVA-Induced AAI

**Figure 2:**
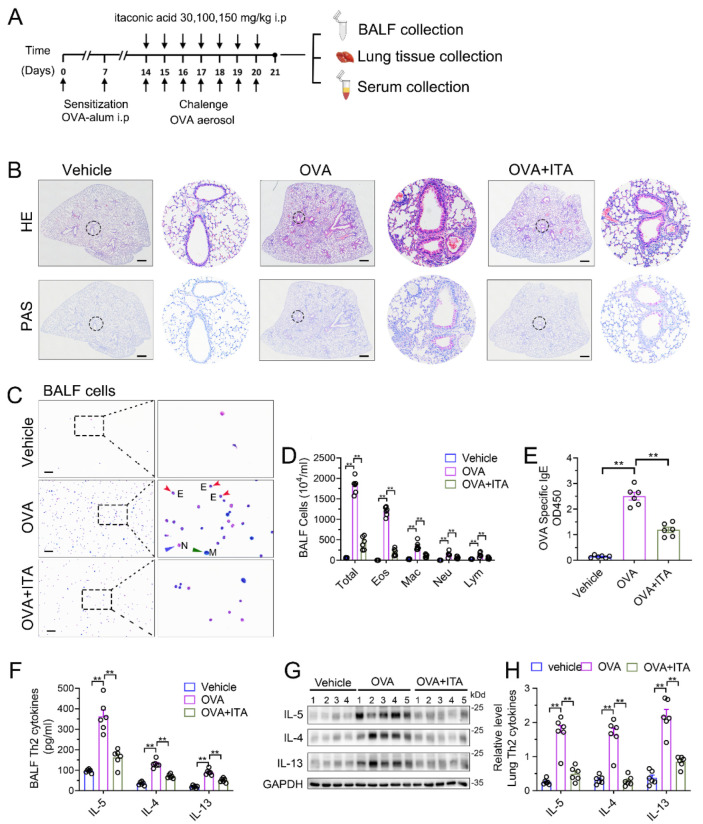
Exogenous itaconate attenuates OVA−induced allergic airway inflammation. (**A**) Protocol for the establishment of OVA-induced allergic airway inflammation and treatment of itaconate. (**B**) Representative whole lung sections stained with HE and PAS, scale bar: 500 μm. A magnified version of the panel can be found in Supplementary File S1: Figure S3. (**C**) Representative images of BALF cells stained with Wright–Giemsa solution. E: red arrows indicate eosinophils, M: green arrow indicates macrophage and N: blue arrow indicates neutrophil. Scale bar: 100 μm. (**D**) The number of total inflammatory cells, eosinophils, macrophages, neutrophils and lymphocytes in BALF. (**E**) The optical density value (OD value) measured at 450 nm represents the level of IgE in serum. (**F**) The level of Th2 cytokines IL-5, IL-4 and IL-13 in BALF. (**G**) Representative blots showing the protein expressions of Th2 cytokines IL-5, IL-4 and IL-13 in lung tissues from vehicle, OVA and OVA+ITA groups. (**H**) Quantification of the expression of IL-5, IL-4 and IL-13 in the lung as referencing to GAPDH. Cropped blots are shown, and Supplementary File S2: Figure S3 presents the full-length blots. Data expressed as means ± SEM (*n* = 6). ** *p* < 0.01.

To assess the role of the increased production of itaconate in AAI, we first administered exogenous itaconate in WT mice (Figure 2A). As shown in Supplementary File S1: Figure S2, itaconate ameliorated the airway inflammation in a dose-dependent manner; therefore, we choose a 100 mg/kg itaconate treatment for all the subsequent animal experiments. In Figure 2B, when treated with itaconate, a reduced peribronchial inflammatory cell infiltration, especially eosinophils in the lung and BALF, was found, and a decreased mucus production was identified as compared with the vehicle-treated mice after the OVA sensitization and challenge (Figure 2B–D). Additionally, the itaconate treatment also significantly decreased the OVA-induced elevation of the IgE level in the serum (Figure 2E) as well as Th2 cytokines’ production in the lung and BALF (Figure 2F–H), respectively. Overall, these data demonstrated that the exogenous supplementation of itaconate ameliorated the OVA-induced type 2 airway inflammation; therefore, the itaconate, which was induced by OVA, may in turn function as a negative regulator to suppress the OVA-induced AAI.

### 3.3. Deletion of Irg1 Augments OVA-Induced AAI

**Figure 3:**
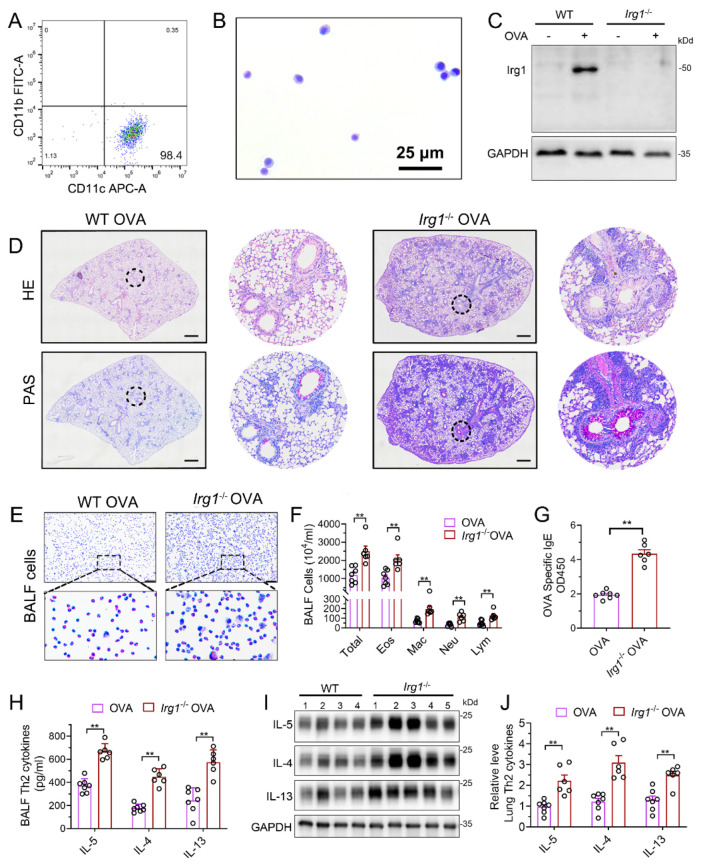
Allergic airway inflammation is augmented in OVA−challenged Irg1^−/−^ mice. (**A**) Representative histogram showing the purity of isolated AMs gated on CD45^+^F4/80^+^CD11b^−^CD11c^+^. (**B**) Representative images of isolated AMs stained with Wright–Giemsa solution. Scale bar: 25 μm. (**C**) Western blot showing Irg1 protein induction in isolated AMs from WT and *Irg1*^−/−^ mice and stimulated with OVA (500 ng/mL) for 24 h. (**D**) Representative whole lung sections stained with HE and PAS; scale bar: 500 μm. (**E**) Representative images of BALF cells stained with Wright–Giemsa solution. Scale bar: 50 μm. (**F**) The number of total inflammatory cells, eosinophils, macrophages, neutrophils and lymphocytes in BALF. (**G**) The optical density value (OD value) measured at 450 nm represents the level of IgE in serum. (**H**) The level of Th2 cytokines IL-5, IL-4 and IL-13 in BALF. (**I**) Representative blots showing the protein expressions of Th2 cytokines IL-5, IL-4 and IL-13 in lung tissues from WT and *Irg1*^−/−^ mice challenged with OVA. (**J**) Quantification of the expression of IL-5, IL-4 and IL-13 in the lung as referencing to GAPDH. Cropped blots are shown, and Supplementary File S2: Figures S4 and S5 present the full-length blots. Data expressed as means ± SEM (*n* ≥ 6). ** *p* < 0.01.

To further confirm the role of itaconate in AAI mice, mice deleted of *Irg1*, the enzyme which expression is reportedly correlated with the intracellular itaconate level [4], was generated (Supplementary File S1: Figure S4A). The homozygous *Irg1*^−/−^ mice were confirmed by PCR (Supplementary File S1: Figure S4B). Additionally, we used primary AMs from the BALF to detect the protein expression of Irg1 in *Irg1*^−/−^ mice. AMs were isolated and the purity was confirmed by flow cytometry (Figure 3A) and Wright Staining (Figure 3B). Western blots demonstrated that OVA markedly induced the Irg1 protein expression in OVA-stimulated AMs, which was absent in *Irg1*^−/−^ AMs (Figure 3C). 

Then, homozygous *Irg1*^−/−^ and the according background WT mice were subjected to the OVA sensitization and challenge. The results demonstrated increased peribronchial inflammatory cell infiltration and airway PAS^+^ staining in *Irg1*^−/−^ mice as compared with the WT allergic controls (Figure 3D). The OVA-induced increase in the number of total BALF inflammatory cells, eosinophils, macrophages, neutrophils and lymphocytes in the WT allergic mice were augmented in *Irg1*^−/−^ mice, while more than 80% of the total BALF inflammatory cells were eosinophils (Figure 3E,F). Similarly, compared to the WT allergic mice, the level of OVA-specific IgE in the serum was further elevated in the OVA-challenged *Irg1*^−/−^ mice (Figure 3G). Moreover, the OVA-challenged *Irg1*^−/−^ mice demonstrated significantly increased Th2 cytokines’ production, including IL-5, IL-4 and IL-13, compared with the OVA-challenged WT mice in the lung and BALF (Figure 3H–J). Collectively, these results demonstrated that *Irg1*^−/−^ mice exhibited increased eosinophilic airway inflammation, mucous cell metaplasia and Th2-mediated immune responses in the OVA-induced AAI, which further confirmed that itaconate was a negative regulator of the OVA-induced AAI.

### 3.4. Itaconate Suppresses Mitochondrial NLRP3 Inflammasome Activation, Oxidative Stress and Mitochondrial Dynamics in AAI

**Figure 4:**
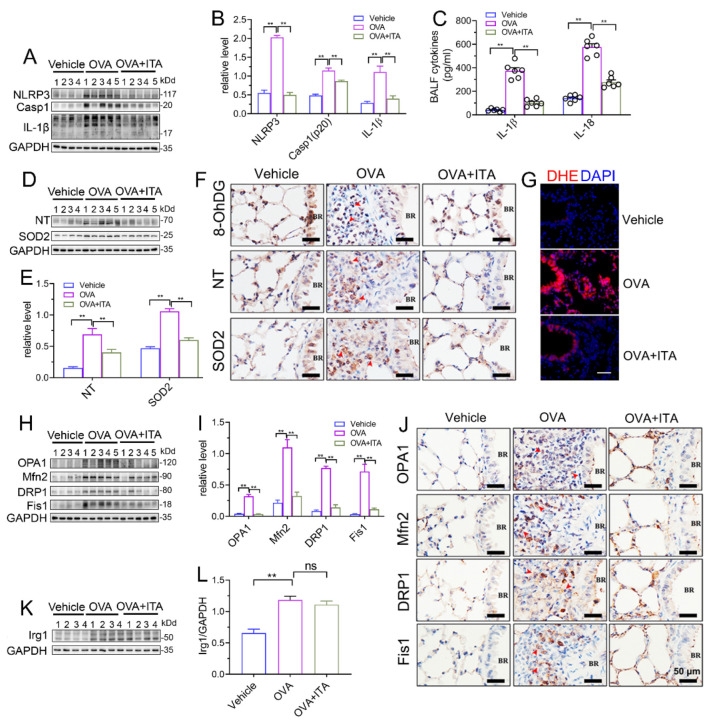
Itaconate suppresses NLRP3 inflammasome activation, oxidative stress and mitochondrial dynamics in allergic airway inflammation. (**A**) Representative blots of NLPR3, Caspase-1 (p20) and IL-1β (p17) in lung tissue. (**B**) Quantification of the expression of NLPR3, Caspase-1 (p20) and IL-1β (p17) in the lung as referencing to GAPDH. (**C**) The level of IL-1β and IL-18 in BALF. (**D**) Representative blots of nitrotyrosine (NT) and SOD2 in lung tissue. (**E**) Quantification of the expression of nitrotyrosine (NT) and SOD2 in the lung as referencing to GAPDH. (**F**) Representative lung sections of the immunohistologic staining of 8-OHdG, nitrotyrosine and SOD2; red arrows indicate the immunopositive cells; scale bar: 50 μm. (**G**) Representative lung sections of DHE staining; scale bar: 50 μm. (**H**) Representative blots of OPA1, Mfn2, DRP1 and Fis1 in lung tissue. (**I**) Quantification of the expression of OPA1, Mfn2, DRP1 and Fis1 in the lung as referencing to GAPDH. (**J**) Representative lung sections of the immunohistologic staining of OPA1, Mfn2, DRP1 and Fis1; red arrows indicate the immunopositive cells; scale bar: 50 μm. (**K**) Representative blots of Irg1 in lung tissue. (**L**) Quantification of the expression of Irg1 in the lung as referencing to GAPDH. Cropped blots are shown, and Supplementary File S2: Figures S6–S9 presents the full-length blots. Data expressed as means ± SEM (*n* = 6). ** *p* < 0.01, ns: no significant difference.

We next aimed to investigate how itaconate exerted its inhibitory effect on AAI. NLRP3 inflammasome and oxidative stress are well characterized to play critical roles in AAI [29]. In our present study, the OVA sensitization and challenge resulted in the activation of the NLRP3 inflammasome and oxidative stress in the murine lung, while the itaconate blocked these OVA-induced changes. Briefly, the itaconate decreased the OVA-induced activation of the NLRP3 inflammasome, as evidenced by reductions of NLPR3, Caspase-1 (p20) and IL-1β (p17) protein expression and IL-1β/IL-18 cytokines’ secretion (Figure 4A–C), and suppressed the OVA-induced oxidative stress, as shown by the decreases in nitrotyrosine, SOD2, 8-OhDG expression and DHE (ROS probe) fluorescence intensity (Figure 4D–G). Similarly, the itaconate treatment inhibited the mitochondrial fusion/fission, as demonstrated by the reduced expressions of OPA1, Mfn2, Drp1 and Fis1 (Figure 4H–J). However, the OVA-induced protein expression of Irg1 was not affected by the itaconate treatment (Figure 4K,L).

**Figure 5:**
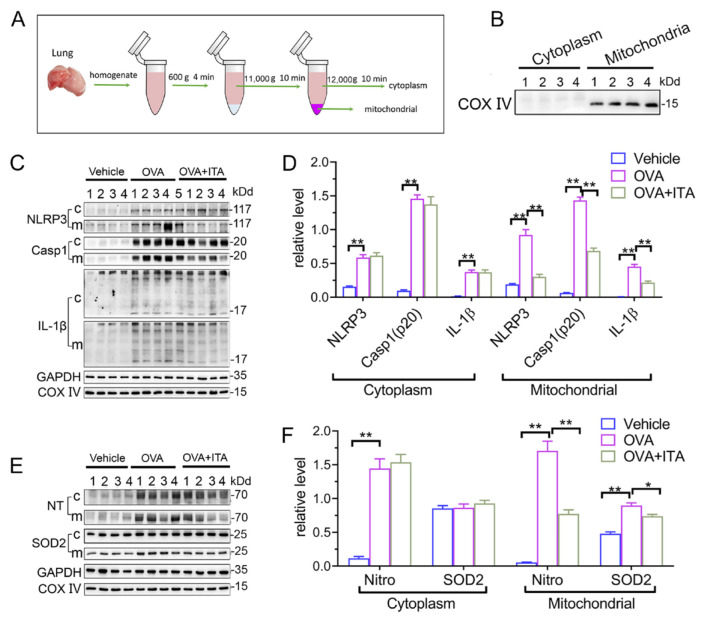
Itaconate suppresses mitochondrial NLRP3 inflammasome activation, oxidative stress and mitochondrial dynamics in allergic airway inflammation. (**A**) Protocol for mitochondrial and cytosolic protein fractionation of lung tissue. (**B**) Representative blots of COX IV in the mitochondrial and cytosolic fractions. (**C**) Representative blots of NLPR3, Caspase-1 (p20) and IL-1β (p17) in cytosolic (c) and mitochondrial (m) fraction of lung tissue. (**D**) Quantitative analysis of NLPR3, Caspase-1 (p20) and IL-1β (p17) in cytosolic and mitochondrial fraction. (**E**) Representative blots of nitrotyrosine (NT) and SOD2 in cytosolic and mitochondrial fraction of lung tissue. (**F**) Quantitative analysis of nitrotyrosine and SOD2 in the mitochondrial and cytosolic fractions. Cropped blots are shown, and Supplementary File S2: Figures S10–S12 presents the full-length blots. Data expressed as means ± SEM (*n* = 6). * *p* < 0.05, ** *p* < 0.01.

It has been suggested that mitochondrial reactive oxygen species (mtROS) have been implicated in NLRP3 activation [30], and the mitochondrial localization of NLRP3 precedes the inflammasome activation [31]. We therefore separated the lung tissue into mitochondrial and cytosolic fractions (Figure 5A), and this separation was successful, as the expression of COX IV was exclusively found in the mitochondrial fraction (Figure 5B). Then, we found that the expression of NLRP3 and mature IL-1β (p17)/Caspase-1 (p20) in the mitochondrial and cytosolic fractions were both significantly increased in OVA-challenged mice (Figure 5C,D). Additionally, the abundance of nitrotyrosine in the mitochondrial and cytosolic fractions was also both significantly increased in OVA-challenged mice, while the expression of SOD2 was only increased in the mitochondrial fraction (Figure 4E,F). However, only the mitochondrial NLRP3 inflammasome activation and mitochondrial oxidative stress were inhibited by the itaconate treatment (Figure 4C–F), suggesting that mitochondria are the primary targets of itaconate when exerting its protective effect on AAI. Consistently, the administration of MitoTEMPO, a mitochondria-targeted ROS scavenger, significantly attenuated the OVA-induced AAI (Supplementary File S1: Figure S5), NLRP3 inflammasome activation, oxidative stress and mitochondrial fusion/fission (Supplementary File S1: Figure S6).

### 3.5. Itaconate Regulates Energy Metabolism and Mitochondrial Morphology in AAI

**Figure 6:**
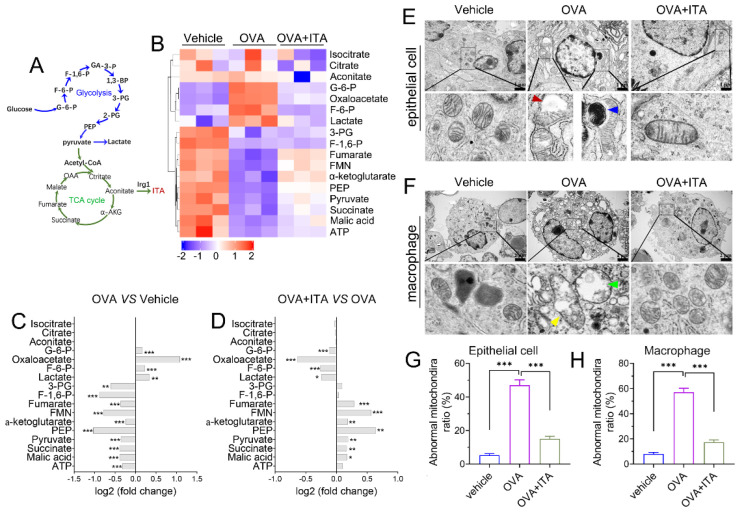
Itaconate regulates energy metabolism and mitochondrial morphology in allergic airway inflammation. (**A**) Schematic overview of metabolites involved in glycolysis and TCA cycle. (**B**) Heatmap showing relative levels of glycolysis and TCA cycle intermediate metabolites in vehicle, OVA and OVA+ITA group (three mice per group). (**C**) Fold change of glycolysis and TCA cycle metabolites in OVA group as compared with vehicle group. (**D**) Fold change of glycolysis and TCA cycle metabolites in OVA+ITA group as compared with OVA group. (**E**) Representative lung section showing mitochondrial morphological changes in epithelial cells. Scale bar: 1 μm. (**F**) Representative lung section showing mitochondrial morphology changes in lung macrophages. Scale bar: 2 μm. (**G**,**H**) The rates of abnormal mitochondria in epithelial cell and macrophage from vehicle, OVA and OVA+ITA group. Red arrowhead indicates cavity formation, blue arrowhead indicates black dense compact, yellow arrowhead indicates crista loss and rupture and green arrowhead indicates swelling. Data expressed as means ± SEM (*n* = 3). * *p* < 0.05, ** *p* < 0.01, *** *p* < 0.001.

We performed targeted organic acid (OA) metabolomics profiling of murine lung tissues from the vehicle, OVA and OVA plus itaconate group to identify changes of energy metabolites (Figure 6A). As compared to the vehicle group, four metabolites were significantly upregulated, including metabolites from glycolysis [glucose 6-phosphate (G-6-P), fructose 6-phosphate (F-6-P) and lactate] and oxaloacetate in the OVA group (Figure 6B,C), while they were markedly decreased in the OVA plus itaconate group (Figure 6B,D). Additionally, ten metabolites were significantly decreased, including seven metabolites from the TCA cycle [pyruvate, α-ketoglutarate, succinate, fumarate, malate, flavin mononucleotide (FMN), adenosine 5′-triphosphate (ATP)] and three metabolites from glycolysis [3-Phospho-D-glycerate (3-PG), Phosphoenolpyruvate (PEP) and Fructose 1,6-biphosphate(F-1,6-P)] in the OVA group in comparison to the vehicle group (Figure 6B, C). However, the itaconate treatment markedly increased these metabolites, except for 3-PG, F-1,6-P and ATP, which were increased but did not reach a statistically significant difference (Figure 6B–D). However, three metabolites from the TCA cycle [isocitrate, citrate and aconitate] were not affected by either the OVA challenge or ITA treatment (Figure 6B–D). To assess whether energy metabolism changes were in parallel with the morphological changes in mitochondria, we examined the ultrastructure of mitochondria using TEM. We found there were mitochondrial structural changes such as swelling, crista loss and ruptures, cavity formation and black dense compact formation in both epithelial cells (Figure 6E) and macrophages (Figure 6F) in an OVA-sensitized and challenged lung compared with the vehicle lung. However, the itaconate treatment improved the morphology of mitochondria to the level comparable to that of the vehicle group (Figure 6E,F). Then, the relative number of abnormal mitochondria was qualified accordingly [32]. The rates of abnormal mitochondria in epithelial cells or macrophages in the OVA group were significantly increased compared with the vehicle group, while the itaconate decreased such increase (Figure 6G,H).

### 3.6. Itaconate Inhibits NLRP3 Inflammasome Activation, Oxidative Stress and Mitochondrial Fusion/Fission in Primary AMs

**Figure 7:**
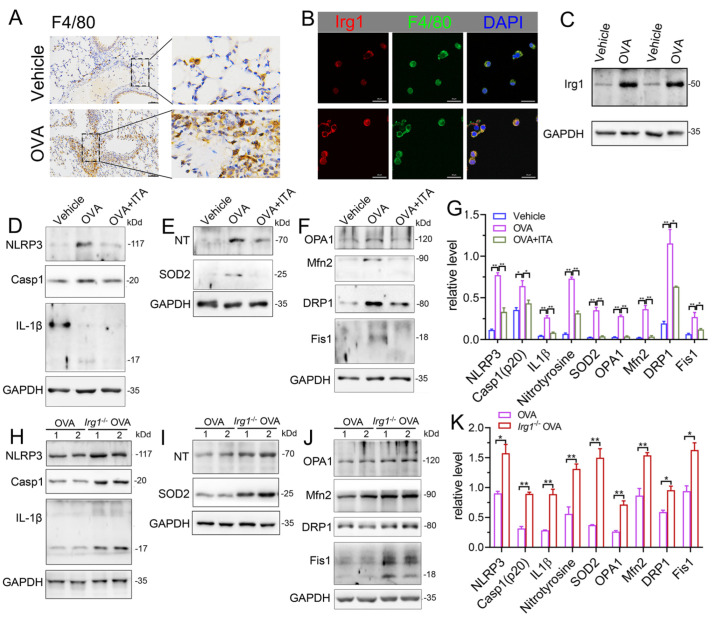
Itaconate inhibits the activation of NLRP3 inflammasome, oxidative stress and mitochondrial fusion/fission in BALF alveolar macrophages. (**A**) Representative lung sections of the immunohistologic staining of F4/80 in vehicle and OVA mice; scale bar: 50 μm. (**B**) Representative double immunofluorescent staining of F4/80 and Irg1 in BALF AMs from vehicle and OVA group. Scale bar: 20 μm. (**C**) Representative blots of Irg1 in BALF AMs from vehicle and OVA mice. (**D**) Representative blots of NLPR3, Caspase-1 (p20) and IL-1β (p17) in BALF AMs from vehicle, OVA and OVA+ITA mice. (**E**) Representative blots of nitrotyrosine (NT) and SOD2 in BALF AMs from vehicle, OVA and OVA+ITA mice. (**F**) Representative blots of OPA1, Mfn2, DRP1 and Fis1 in BALF AMs from vehicle, OVA and OVA+ITA mice. (**G**) Quantitative analysis of NLPR3, Caspase-1 (p20), IL-1β (p17), nitrotyrosine (NT), SOD2, OPA1, Mfn2, DRP1 and Fis1 in AMs from vehicle, OVA and OVA+ITA group. (**H**) Representative blots of NLPR3, Caspase-1 (p20) and IL-1β (p17) in BALF AMs from OVA and *Irg1*^−/−^ OVA mice. (**I**) Representative blots of nitrotyrosine (NT) and SOD2 in BALF AMs from OVA and *Irg1*^−/−^ OVA mice. (**J**) Representative blots of OPA1, Mfn2, DRP1 and Fis1 in BALF AMs from OVA and *Irg1*^−/−^ OVA mice. (**K**) Quantitative analysis of NLPR3, Caspase-1 (p20), IL-1β (p17), nitrotyrosine (NT), SOD2, OPA1, Mfn2, DRP1 and Fis1 in AMs from vehicle, OVA and OVA+ITA group. For Western blot, *n* = 3 per group, each replicate pooled cells from three to five mice in vehicle and OVA+ITA group, while in OVA and *Irg1*^−/−^ OVA group, each replicate represented one mouse; before seeding, the cell number was counted to ensure that the adherent AMs number will be no less than 2.5 × 10^5^ cells/well. Cropped blots are shown, and Supplementary File S2: Figures S13–S23 presents the full-length blots. * *p* < 0.05, ** *p* < 0.01.

Macrophages play a key role in innate immunity. Itaconate is the most abundant metabolite in LPS-treated macrophages, and is synthesized by the enzyme encoded by *Irg1* [17]. In the OVA-induced AAI, macrophages were significantly infiltrated in the lung (Figure 7A). The protein expression of Irg1 was elevated in BALF AMs from OVA-challenged mice as compared to that from vehicle mice, as evidenced by the immunofluorescence (Figure 7B) and immunoblot (Figure 7C), demonstrating that the Irg1/itaconate pathway is involved in AMs’ immune response in AAI. Therefore, we isolated BALF AMs from mice of the vehicle, OVA and OVA plus itaconate group. The results demonstrated that the expression levels of NLRP3, active caspase-1 (p20) and mature IL-1β (p17) were significantly increased in BALF AMs from the OVA group (Figure 7D,G), indicating the activation of the NLRP3 inflammasome. Additionally, the expression levels of nitrotyrosine and SOD2, which represent markers of the NO-dependent oxidative stress and anti-oxidative system, respectively, were also significantly increased in BALF AMs from the OVA group (Figure 7E,G). Moreover, significantly increased expression levels of OPA1, Mfn2, Drp1 and Fis1, which are markers of mitochondrial fusion/fission, were demonstrated in BALF AMs from the OVA group (Figure 7F,G). However, the elevated expression levels were not observed in BALF AMs from the OVA plus itaconate group (Figure 7D–G). Furthermore, to confirm the effect of endogenous itaconate on AMs’ immune responses, we isolated AMs from mice of the WT OVA and *Irg1*^−/−^ OVA group. The results demonstrated that the OVA-induced activation of the NLRP3 inflammasome (Figure 7H,K), oxidative stress (Figure 7I,K) and mitochondrial fusion/fission (Figure 7J,K) in BALF AMs was further augmented when *Irg1* was deleted. These data suggested that both the exogenous and endogenous itaconate regulated the NLRP3 inflammasome activation, oxidative stress and mitochondrial fusion/fission of AMs in AAI. 

## 4. Discussion

Itaconate, an endogenous metabolite from cis-aconitic acid decarboxylation, has been suggested as a critical molecule in the field of immunometabolism in the last few years, and it is involved in the metabolic reprogramming of immune cells and exerts anti-inflammatory effects in macrophages and dendritic cells [5,33,34]. As highly variable endogenous levels of itaconate have been found, itaconate is presumed to be involved in several diseases [6,7,8,9,10,11]. Although the itaconate level was clearly increased in bone marrow-derived dendritic cells (BMDCs) pulsed with allergen HDM and STING or HDM alone [13], its level in the allergic lung is unknown. Our present study revealed that the level of itaconate was notably increased in the OVA-challenged lung; correspondingly, lungs from the OVA-challenged group demonstrated a significant induction of Irg1 mRNA and protein expression, suggesting that the elevated Irg1 expression and its derived itaconate production are involved in AAI. At present, itaconate is considered to be synthesized mainly in immune cells such as macrophages. Macrophages are activated by many factors, including LPS [4], as well as by ligands of the toll-like receptor (TLR) [35,36] and STING pathway [37]. These stimuli during inflammatory diseases may increase Irg1 to produce itaconate [6,7,8,9], and are therefore potential causes of increased endogenous levels of itaconate in human diseases. However, due to the uncertainty as to how exogenous itaconate passes the plasmamembrane, derivatives of itaconate, especially 4-OI, are widely used in the field of itaconate biology. It has been demonstrated that 4-OI can inhibit the airway inflammation caused by respiratory virus infection [12] or allergen inhalation [13,14]. Nevertheless, whether 4-OI is converted into intracellular itaconate is controversial; Swain et al. report that 4-OI does not yield intracellular itaconate accumulation [38], while Hooftman et al. demonstrated that ^13^C_5_-labeled OI is converted into ^13^C_5_ itaconate intracellularly [18]. Despite this controversy, it is acknowledged that 4-OI induces a strong electrophilic stress response in contrast to itaconate, and therefore its ability to impact certain pathways (such as the ATF3-IKBζ axis) is higher than itaconate [18,38,39]. Therefore, results obtained with 4-OI may not represent the physiological effect of itaconate. In the present study, unmodified natural itaconate, which has been reported to readily enter cells [38], was administrated to OVA-challenged mice. The results demonstrated that the unmodified itaconate significantly ameliorated the airway inflammation, mucus secretion and Th2 cytokine production. Thus, the exogenous administration of unmodified natural itaconate is capable of suppressing AAI. Then, we aimed to investigate whether the endogenous itaconate contributed to AAI. Using mice lacking *Irg1* to establish the murine AAI model, we found that the lack of *Irg1* resulted in an augmented eosinophilic airway inflammation and Th2 immune response to the OVA allergen. Collectively, these results provide evidence that itaconate production is significantly increased in AAI, adding exogenous itaconate suppressed features of AAI, while blocking endogenous itaconate using the *Irg1*^−/−^ mice augmented features of AAI. Therefore, the OVA-induced upregulation of itaconate may be an endogenous protective response to combat the airway inflammation. Our data highlighted the protective role of the endogenous/unmodified natural itaconate in AAI. 

Given the significant protective role of itaconate in AAI, we asked how it exerts the inhibitory effect on AAI. Itaconate and its derivative 4-OI reportedly induce anti-inflammatory effects via mechanisms including specifically blocking the NLRP3 inflammasome activation [18,40], or targeting GAPDH to inhibit the aerobic glycolysis [41]. Moreover, they reportedly demonstrate a potent antioxidant effect through mechanisms including inhibiting the SDH-mediated intracellular mtROS generation [5], and promoting Nrf2 to induce the transcription of the antioxidant genes [17]. Both NLRP3 inflammasome activation and oxidative stress are well-characterized features in AAI [29]. In our present study, the treatment of OVA-challenged mice with itaconate blocked the activation of the NLRP3 inflammasome, suppressed oxidative stress and inhibited mitochondrial fusion/fission, which may underpin the protective effect of itaconate on AAI. 

Furthermore, we noted that the mitochondrial localization of NLRP3 [23,31] has been reported to immediately recognize the mitochondrial damage signals such as mtROS and mtDNA, which are sufficient for the NLRP3 inflammasome activation [30]. Therefore, the positioning of NLRP3 on mitochondria is considered to precede the inflammasome activation. We therefore separated the lung tissue into mitochondrial and cytosolic fractions, and found that theNLRP3 inflammasome activation and oxidative stress in the mitochondrial and cytosolic fractions were both significantly increased in OVA-challenged mice. However, only the mitochondrial NLRP3 inflammasome and mitochondrial oxidative stress were inhibited by the itaconate treatment. Consistently, a mitochondria-targeted ROS scavenger, MitoTEMPO, replicated the effect of itaconate, which significantly attenuated the OVA-induced AAI, NLRP3 inflammasome activation, oxidative stress, as well as mitochondrial fusion and fission. These results suggested that mitochondria are the primary targets of itaconate to exert the protective effect on AAI.

Itaconate is generated in the mitochondrial matrix [4]; it competitively inhibits the succinate dehydrogenase to regulate the TCA cycle [42]. In asthmatic mice, changes in the TCA cycle intermediate levels have been reported. For example, the plasma organic acid (OA) metabolic profiling analysis reveals that among the detected 7 OAs in the TCA cycle, pyruvate, succinate, fumarate, α-KG, malate and citrate were reduced, while lactate was increased [43]. In a similar fashion, succinate and fumarate were depleted in the urine from guinea pigs of experimental asthma [44]. Notably, itaconate has reportedly increased the intracellular levels of succinate, α-KG and glutamate but significantly decreased pyruvate, citrate and malate in cultured neurons [45]. In agreement with these reports, the reduction in TCA cycle OAs but elevation in the lactate level was observed currently in OVA-challenged lungs compared to vehicle lungs. However, itaconate reversed these changes. In particular, the reduction of intermediate OAs in the TCA cycle and elevated lactate level in OVA-challenged mice were similar to previous results observed in asthmatic patients [46,47,48], suggesting that they could serve as biomarkers of asthma. Our present study demonstrated disturbances of OAs’ metabolism in AAI, and provided evidence demonstrating that itaconate exerted a direct impact on the TCA cycle metabolism and glycolysis in AAI, which may be beneficial for improving energy metabolism.

We then asked whether energy metabolism changes were in parallel with mitochondrial morphological changes in AAI, and whether they can be modulated by itaconate. Changes in the mitochondrial structure known as mitochondrial dynamics involving size, shape, activity, trafficking and inter-organelle interactions occur depending on the cell’s energy requirements and pathological cellular process, and impact many cell signaling pathways including bioenergetics [49] and immune responses [50]. The pathophysiology of asthma has been reported to be tightly associated with mitochondrial dysfunction; for example, airway epithelial cells and smooth muscle cells of asthmatic patients show mitochondrial fragmentation and swelling [51,52] and in vivo animal models suggest disturbed mitochondrial function [53,54]. Consistently, we observed more structural changes such as swelling, crista loss and ruptures, cavity formation and black dense compact formation in mitochondria of the epithelial cells and macrophages in OVA-challenged lungs than in vehicle lungs. However, the itaconate treatment inhibited these pathological changes of mitochondria. Taken together, our results suggested that structural changes and the resulting metabolic dysfunction were associated with allergic asthma, and the protective effect of itaconate on AAI may be mediated by improving the mitochondrial metabolic function.

Next, we further investigated which cell type was involved in the protective role of itaconate in AAI. Evidence from BMDCs suggested that the Irg1/itaconate pathway in DCs contributes to the resolution of HDM-induced type 2 airway inflammation [13]. However, as in BALF, more than 90% of cells are AMs [55,56]; their role in the pathogenesis of allergen-induced asthma has attracted more and more attention [16]. AMs’ activation occurs immediately after the antigen challenge, which triggers the release of ROS and thereby resulting in an allergic airway reaction [57]. Moreover, NLRP3 inflammasome activation, which is critical in the pathogenesis of asthma, is triggered only in AMs from HDM-challenged mice, indicating that the activation of the NLRP3 inflammasome in the lungs of asthmatic mice is dependent on AMs [58]. Additionally, the key synthesis enzyme for itaconate, Irg1, is mainly expressed in activated immune cells and strongly induced in LPS-activated macrophages [15]. Itaconate and its derivatives have been reported to regulate macrophages such as BMDM immune responses in vitro [5,17]. In the present study, we confirmed that macrophages significantly infiltrated the lung in response to the OVA challenge, and BALF AMs from OVA-challenged mice demonstrated an elevated Irg1 expression as compared to that from the vehicle mice, indicating that the Irg1/itaconate pathway in AMs was involved in the OVA-induced AAI. Consequently, the effect of itaconate on primary AMs’ immune responses in AAI was studied. We revealed that the exogenous itaconate significantly suppressed the OVA-induced activation of the NLRP3 inflammasome, oxidative stress and mitochondrial fusion/fission in BALF AMs from WT mice, while in AMs from *Irg1*^−/−^ mice, the OVA-induced activation of the NLRP3 inflammasome, oxidative stress and mitochondrial fusion/fission were further augmented. These data suggested that both exogenous and endogenous itaconate directly regulated AMs’ immune responses in allergic airways. 

## 5. Conclusions

Our findings provide evidence for the first time by demonstrating that in AAI, the endogenous itaconate level in the lung is markedly increased, which directly regulates the NLRP3 inflammasome activation and oxidative stress in AMs, indicating that the Irg1/itaconate pathway in AMs is a potential anti-inflammatory and anti-oxidative therapeutic target for the treatment of AAI. Moreover, in the present study, we propose that the unmodified natural itaconate is capable of protecting against AAI, and highlighted that such a protective effect of itaconate may be attributed to its suppression of inflammatory mitochondrial responses, such as the mitochondrial NLRP3 inflammasome activation, mitochondrial oxidative stress and mitochondrial metabolic dysfunction, which extend previous knowledge on the anti-inflammatory and anti-oxidative mechanism of itaconate. 

## Data Availability

Data is contained within the article and Supplementary Material.

## Supplementary Materials

The following supporting information can be downloaded at: https://www.mdpi.com/article/10.3390/antiox12020489/s1, Supplementary File S1: Figure S1. OVA-induced allergic airway inflammation. Representative whole lung sections stained with HE and PAS, scanned by ×10 objective; Figure S2. Itaconate (ITA) dose dependently ameliorates OVA-induced allergic airway inflammation; Figure S3. Representative whole lung sections stained with HE and PAS, scale bar: 500 μm; Figure S4. *Irg1*^−/−^ mice available from Cyagen Biosciences Inc. (Stock# S-KO-02680, Suzhou, China); Figure S5. MitoTEMPO attenuates OVA-induced allergic airway inflammation; Figure S6. MitoTEMPO suppresses NLRP3 inflammasome activation, oxidative stress and mitochondrial dynamics in allergic airway inflammation. Supplementary File S2: Figure S1. Raw blots of Figure 1E; Figure S2. Raw blots of Figure 1L; Figure S3. Raw blots of Figure 2G; Figure S4. Raw blots of Figure 3C; Figure S5. Raw blots of Figure 3I; Figure S6. Raw blots of Figure 4A; Figure S7. Raw blots of Figure 4D; Figure S8. Raw blots of Figure 4G; Figure S9. Raw blots of Figure 4I; Figure S10. Raw blots of Figure 4K; Figure S11. Raw blots of Figure 4L; Figure S12. Raw blots of Figure 4N; Figure S13. Raw blots of Figure 6C; Figure S14. Raw blots of Figure 6D; Figure S15. Raw blots of Figure 6E; Figure S16. Raw blots of Figure 6F; Figure S17. Raw blots of Figure 6G; Figure S18. Raw blots of Figure 6H; Figure S19. Raw blots of Figure 6I; Figure S20. Additional bands used for statistics in Figure 6D; Figure S21. Additional bands used for statistics in Figure 6E; Figure S22. Additional bands used for statistics in Figure 6F; Figure S23. Additional bands used for statistics in Figure 6G–I; Figure S24. Raw blots of Figure S5G; Figure S25. Raw blots of Figure S6A; Figure S26. Raw blots of Figure S6E; Figure S27. Raw blots of Figure S6J.
