# Supplementary material for: Itaconate Suppresses the Activation of Mitochondrial NLRP3 Inflammasome and Oxidative Stress in Allergic Airway Inflammation
Source: Antioxidants (Basel). 2023 Feb 15;12(2):489. doi: 10.3390/antiox12020489 (PMC9951851; doi:10.3390/antiox12020489)
Supplement: Supplementary file 1 [file antioxidants-12-00489-s001.zip › antioxidants-2129788-original images.pdf]

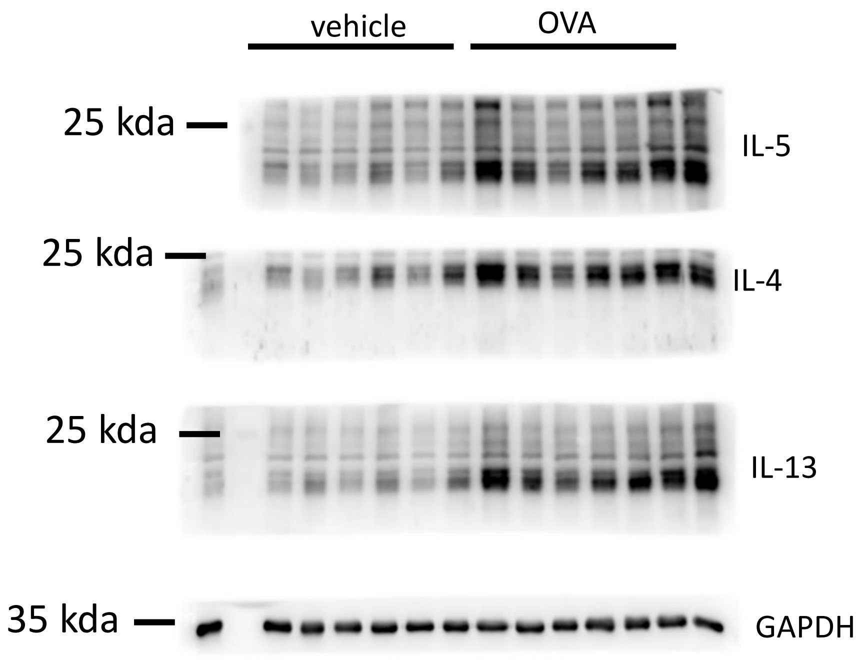

Figure S1. Raw blots of Figure 1E

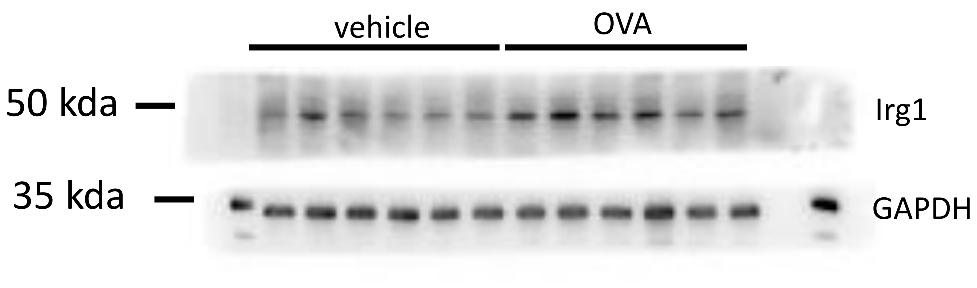

Figure S2. Raw blots of Figure 1L

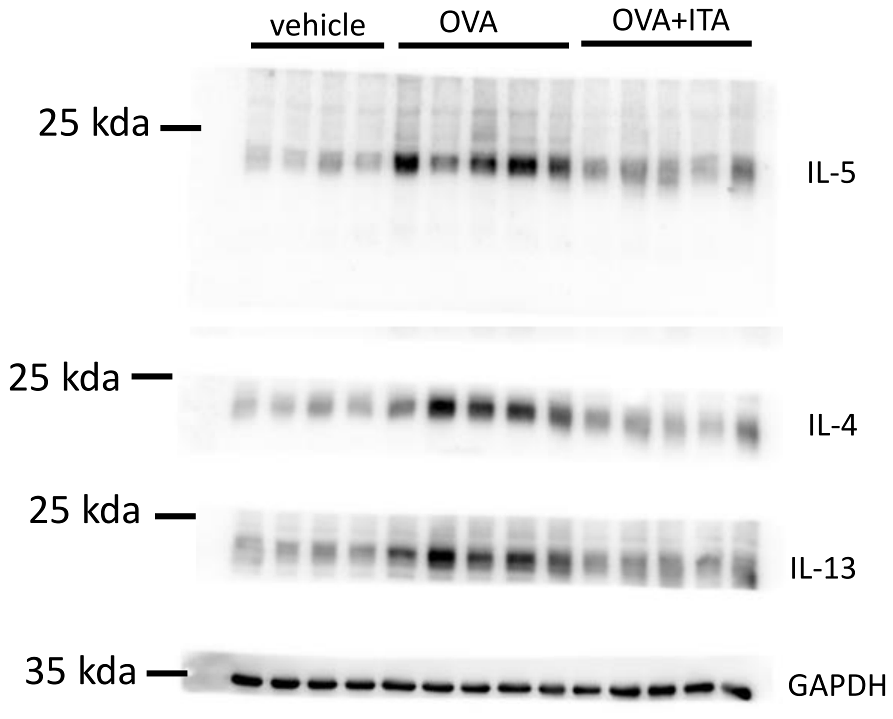

Figure S3. Raw blots of Figure 2G

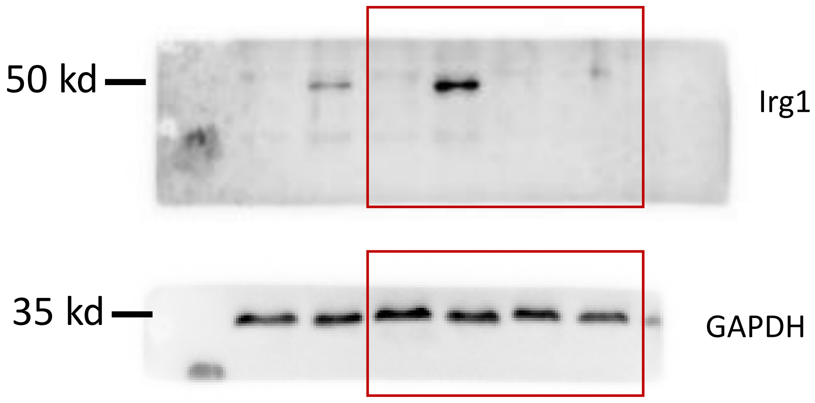

Figure S4. Raw blots of Figure 3C

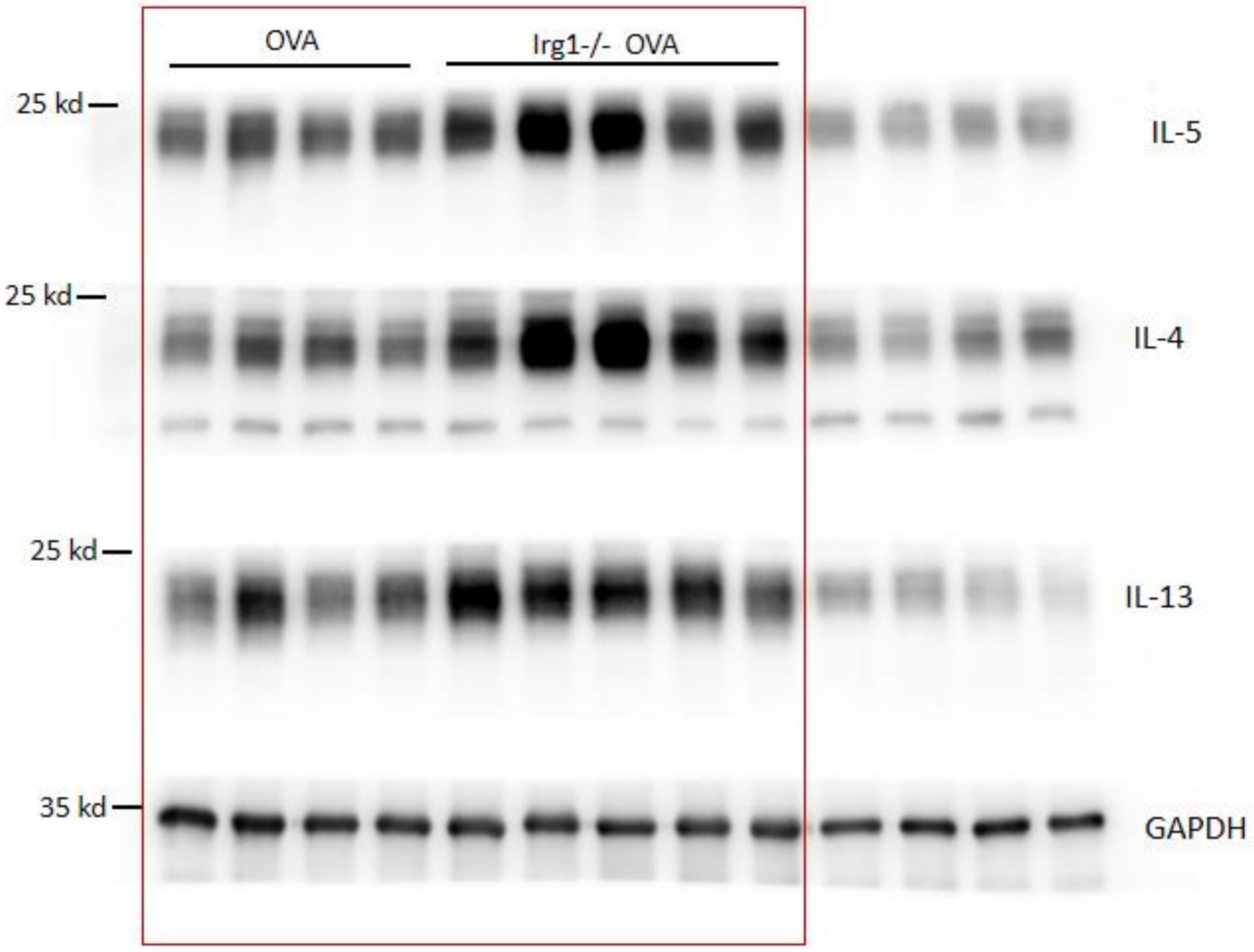

Figure S5. Raw blots of Figure 3I

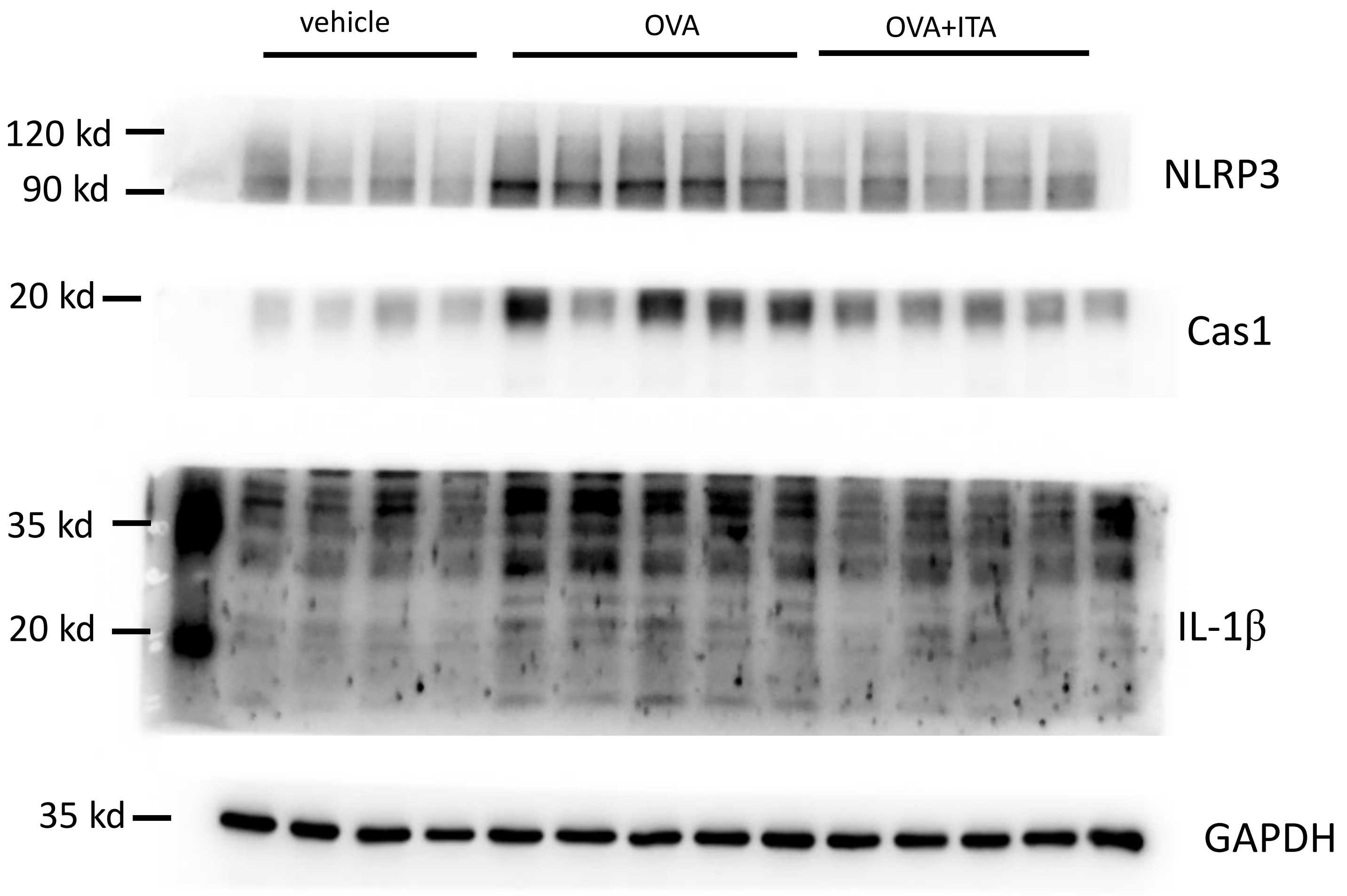

Figure S6. Raw blots of Figure 4A

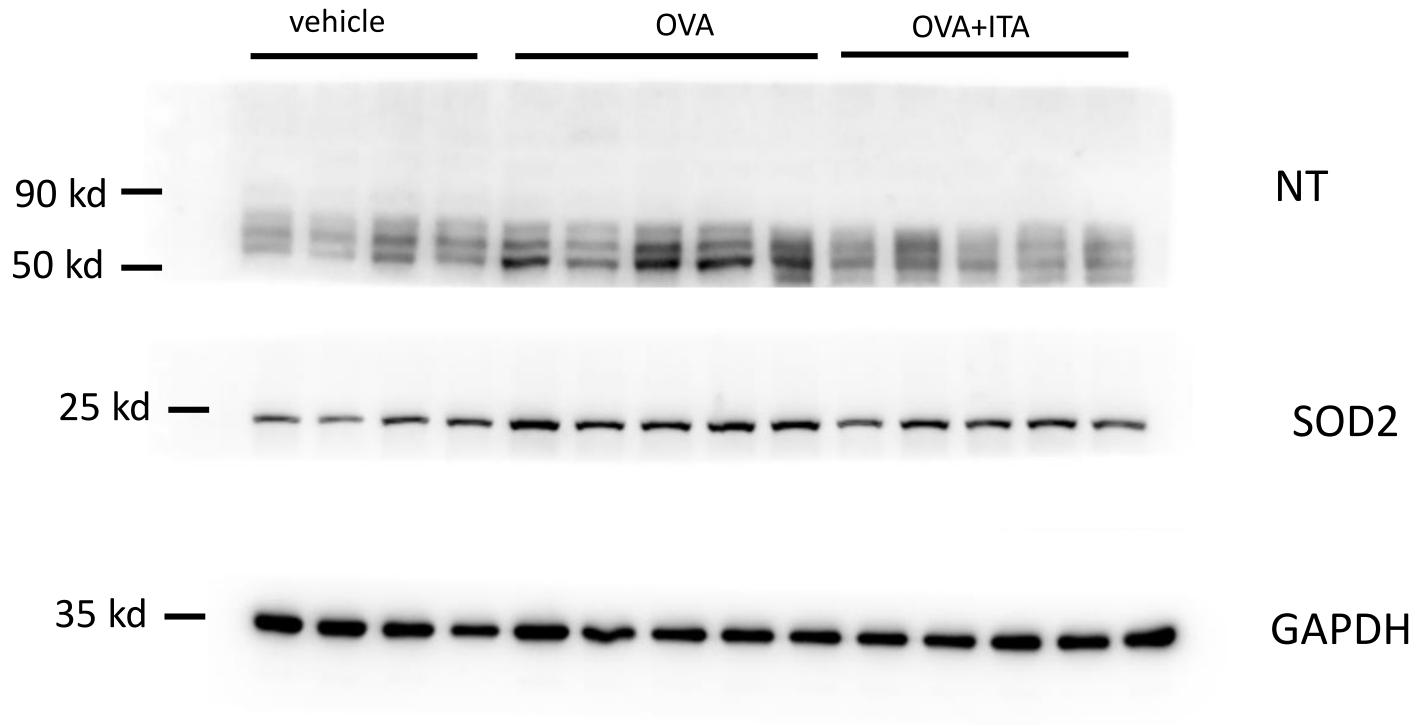

Figure S7. Raw blots of Figure 4D

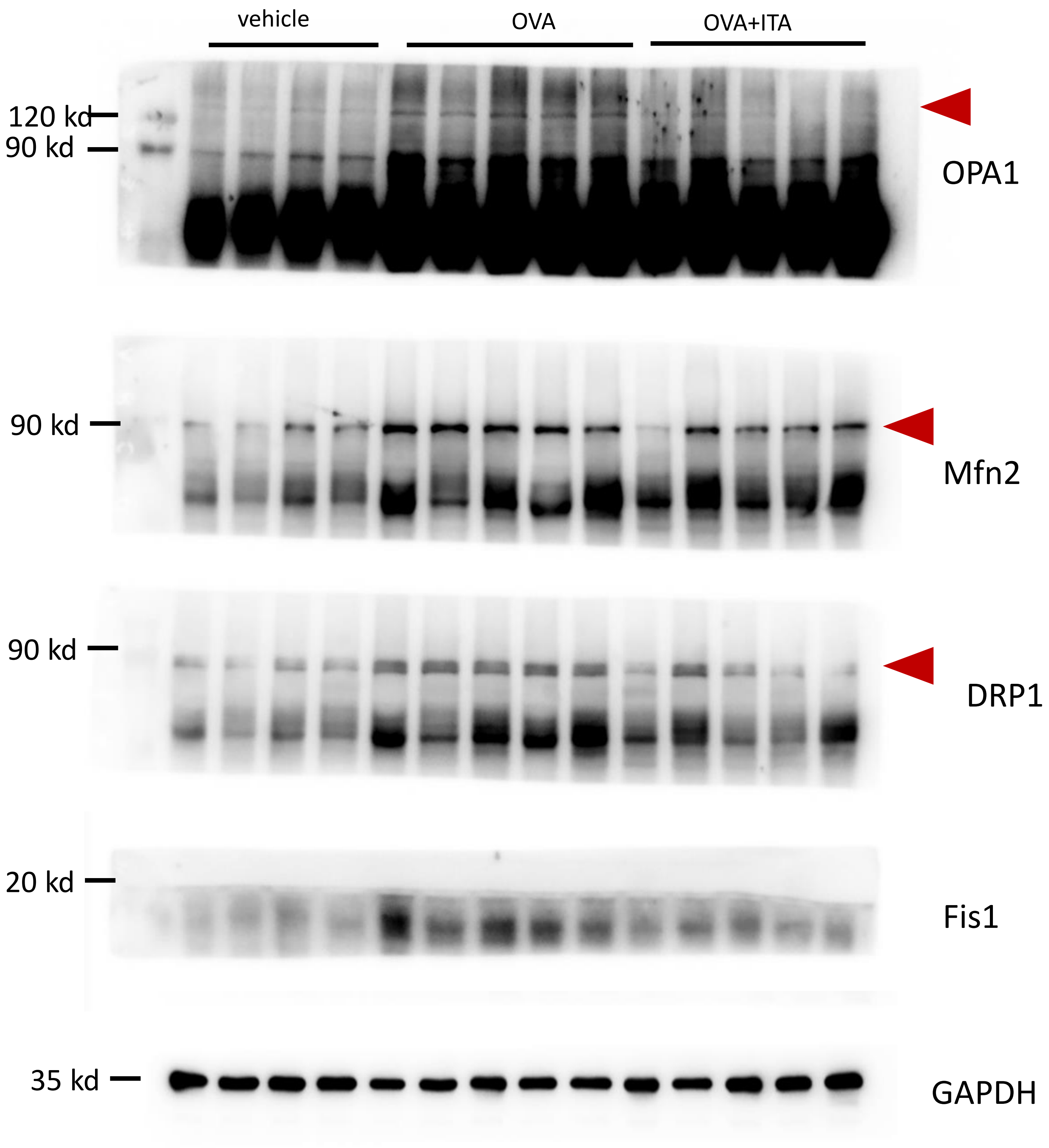

Figure S8. Raw blots of Figure 4G

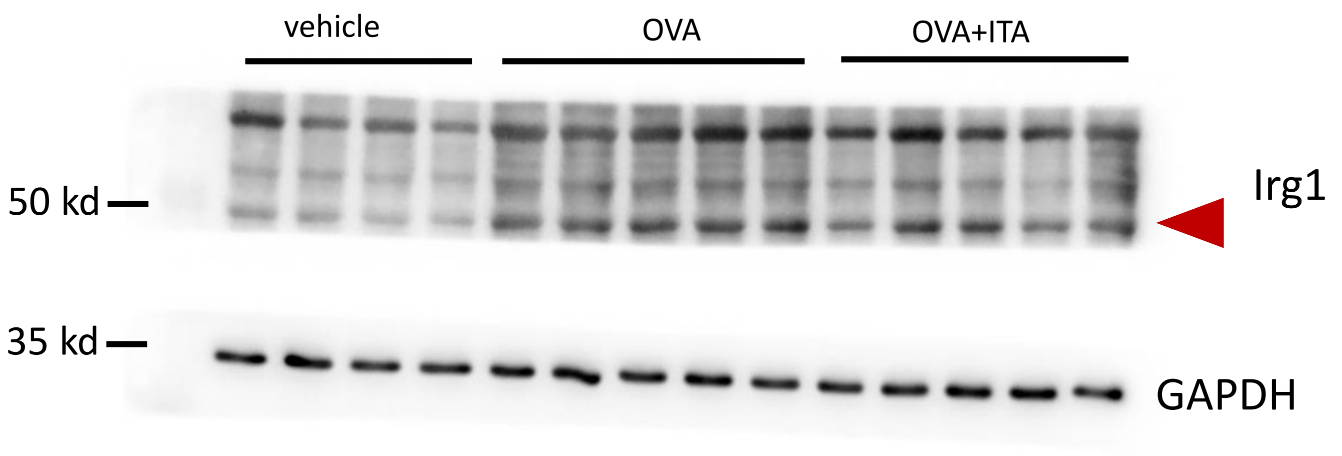

Figure S9. Raw blots of Figure 4I

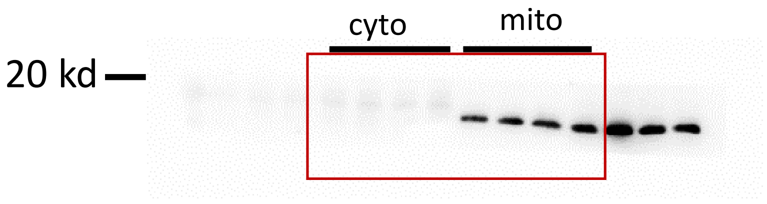

Figure S10. Raw blots of Figure 4K

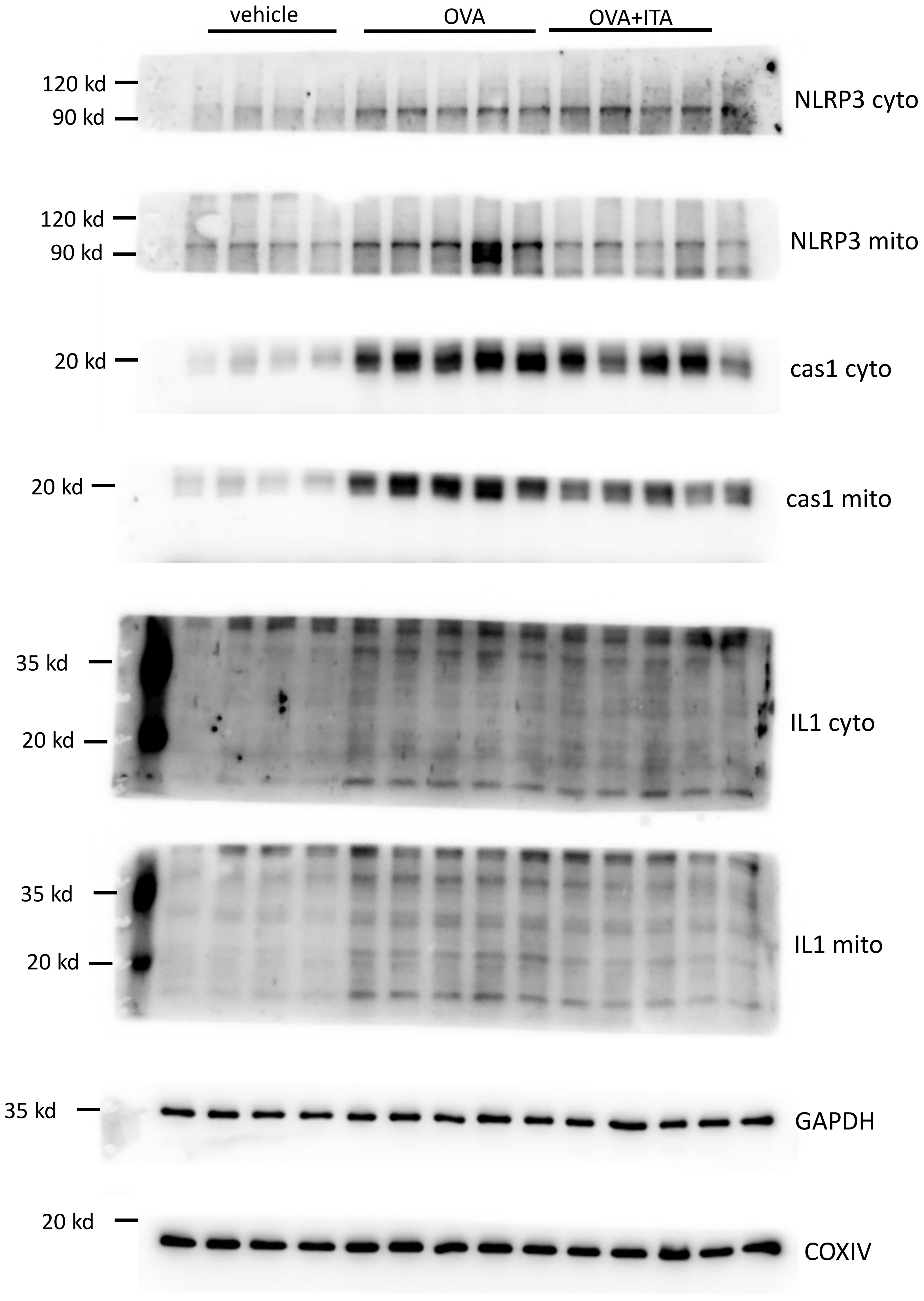

Figure S11. Raw blots of Figure 4L

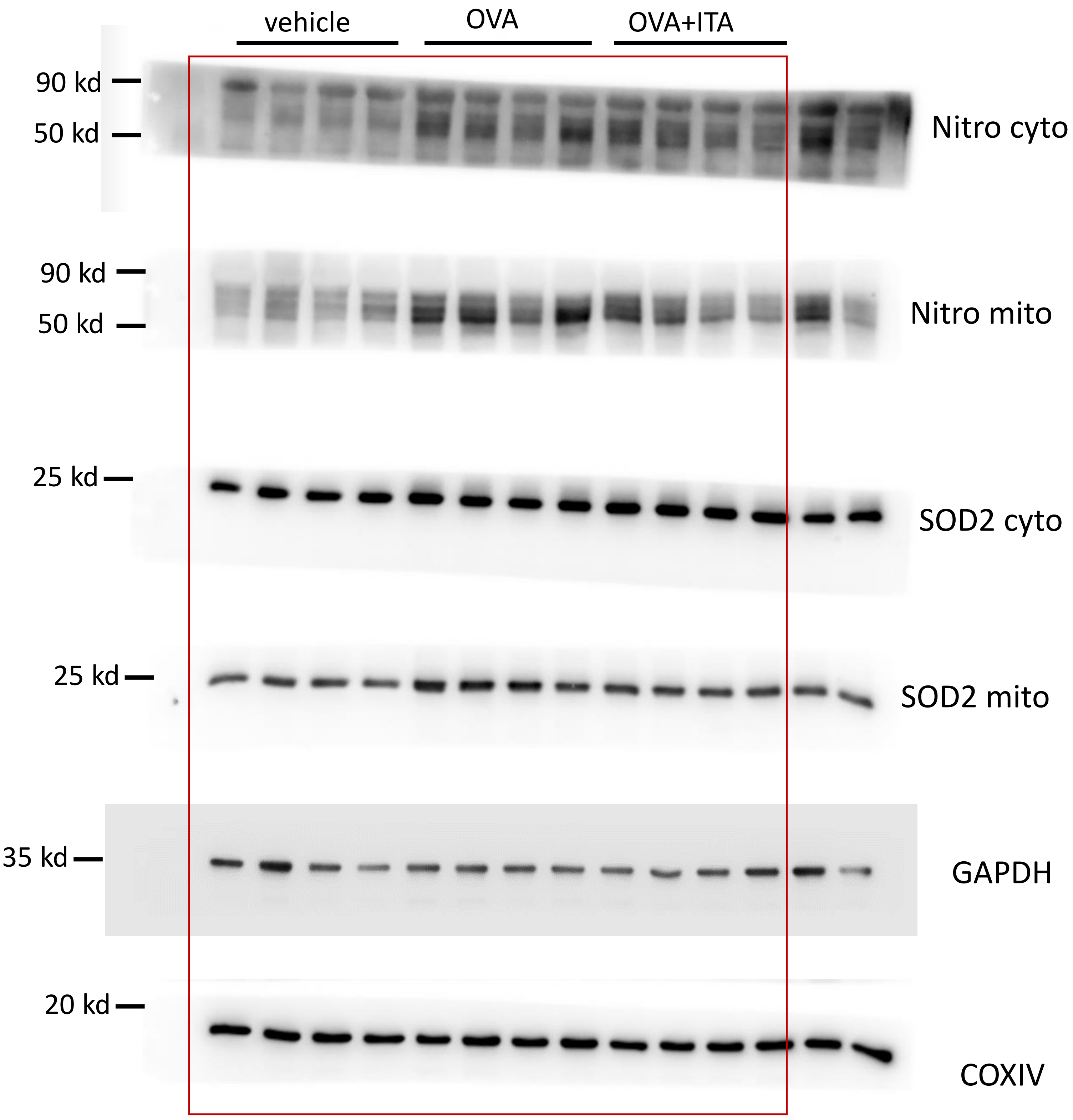

Figure S12. Raw blots of Figure 4N

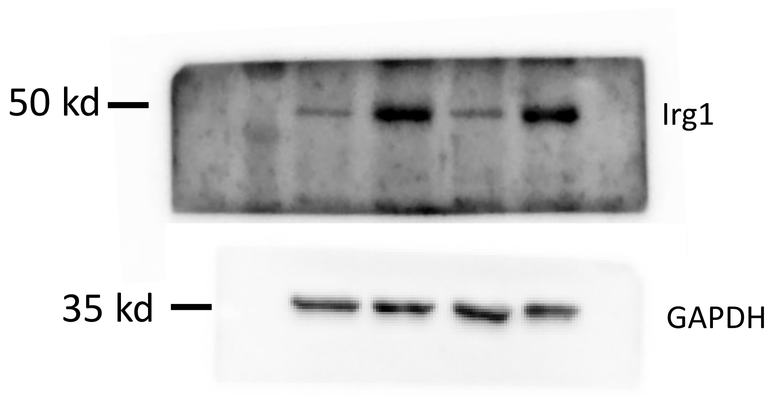

Figure S13. Raw blots of Figure 6C

A

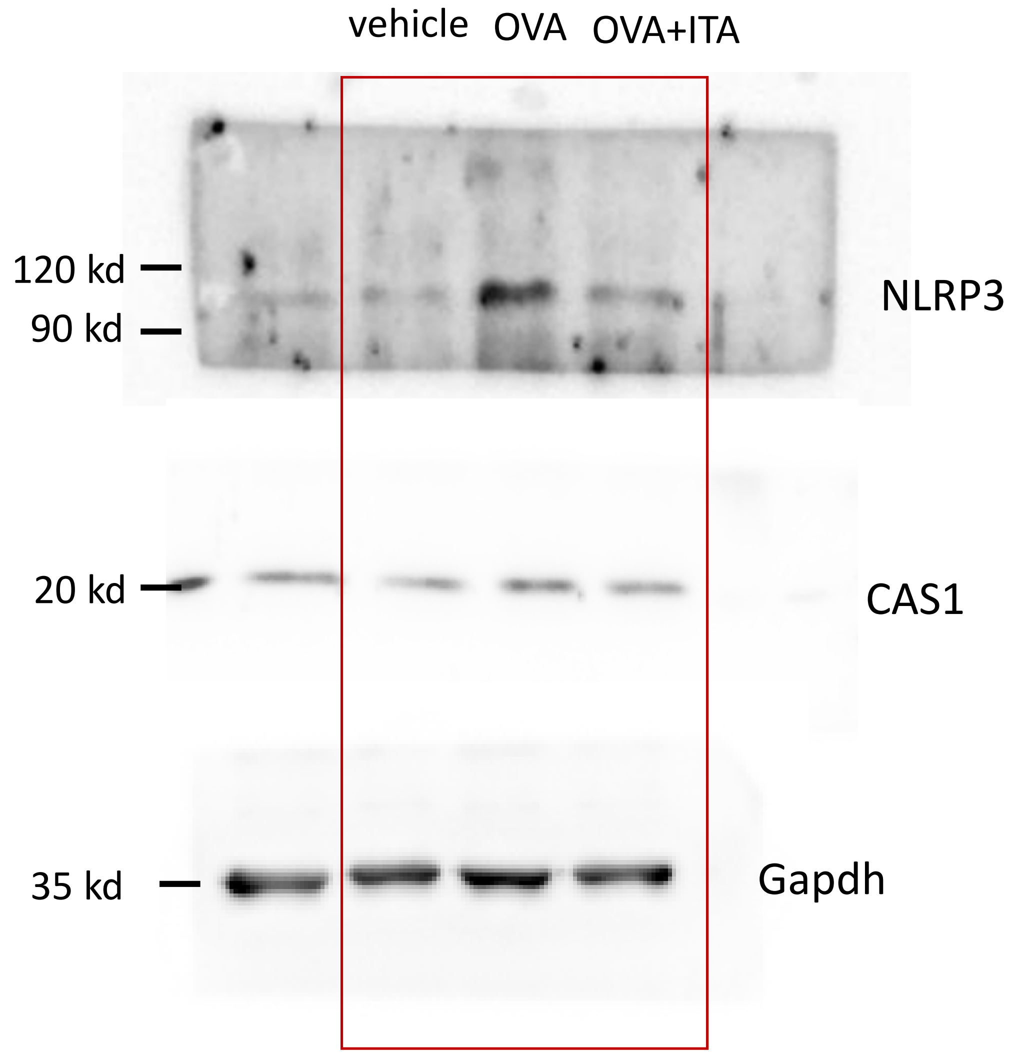

B

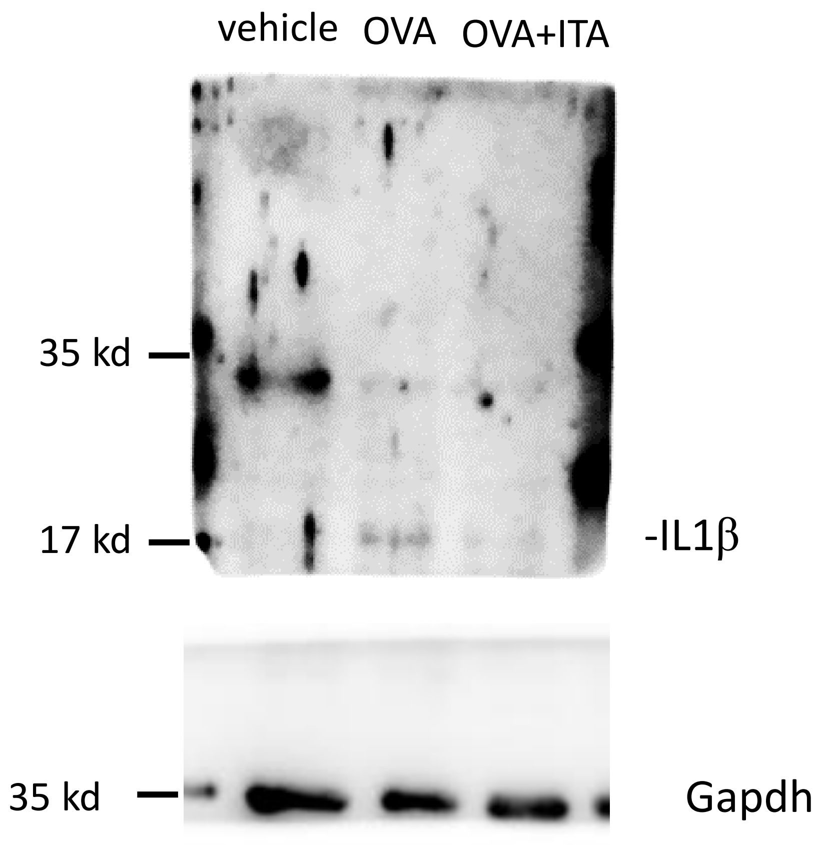

Figure S14. Raw blots of Figure 6D

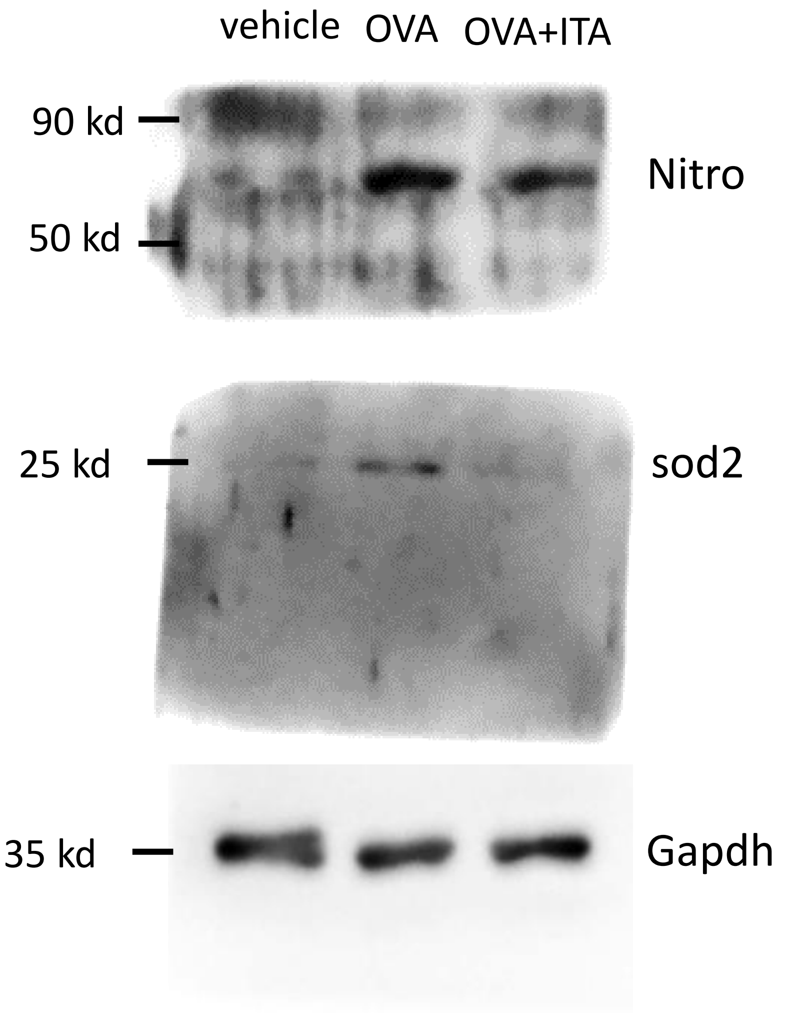

Figure S15. Raw blots of Figure 6E

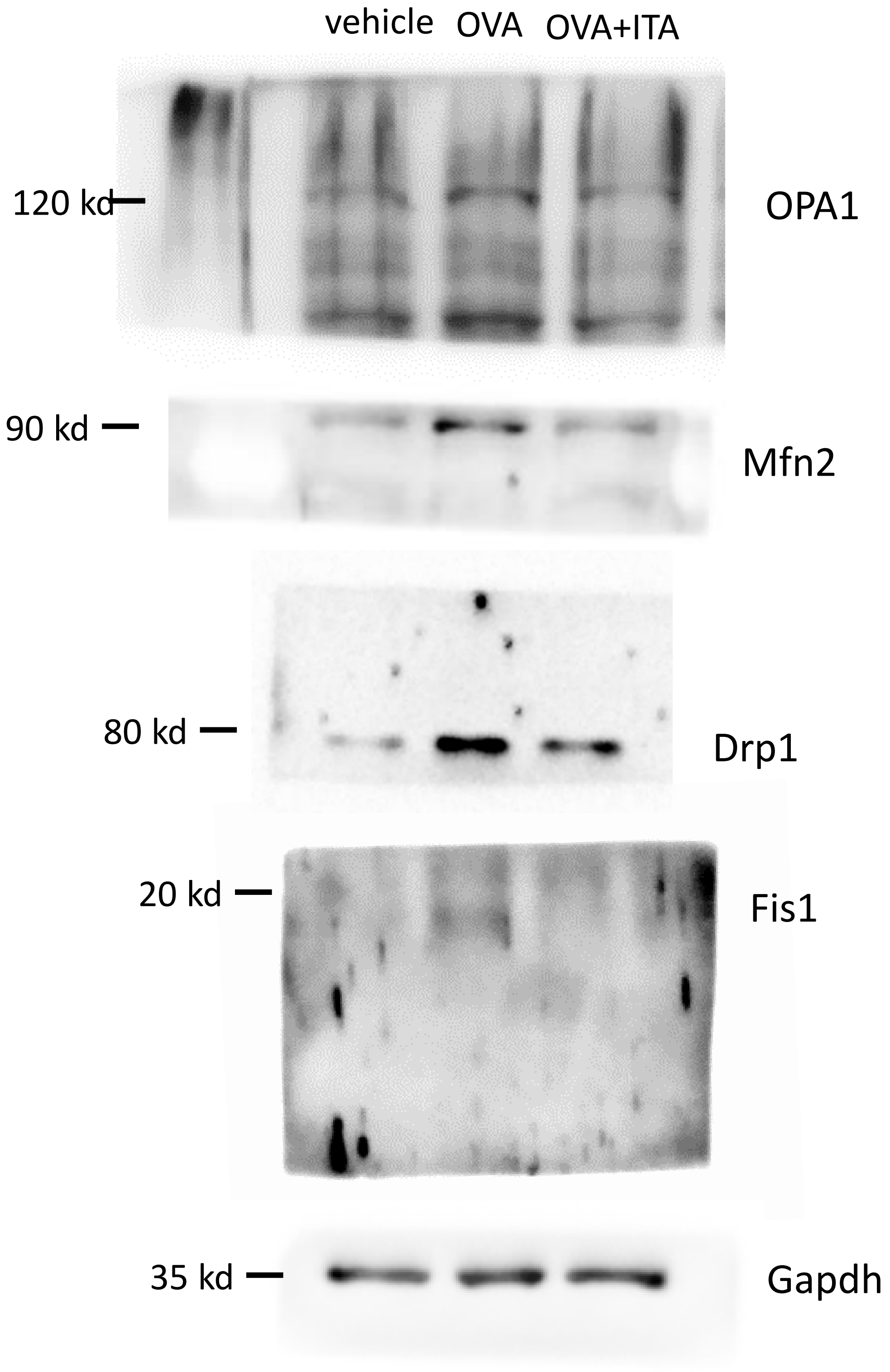

Figure S16. Raw blots of Figure 6F

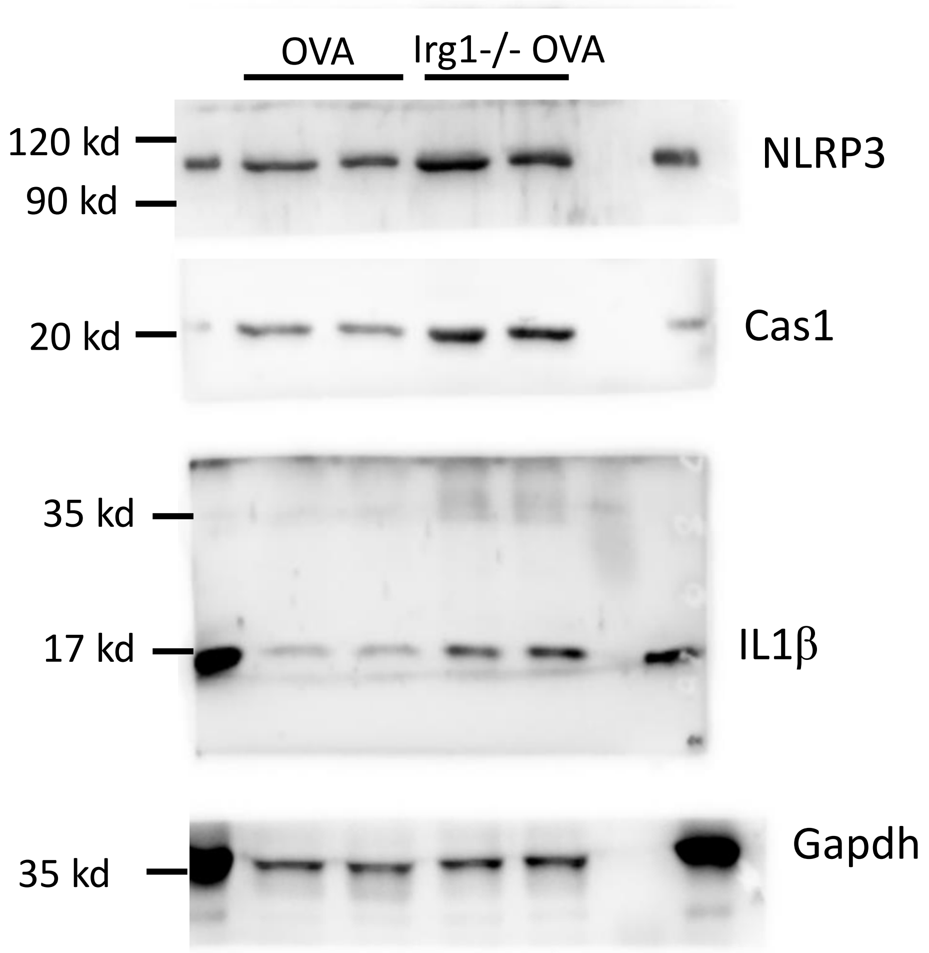

Figure S17. Raw blots of Figure 6G

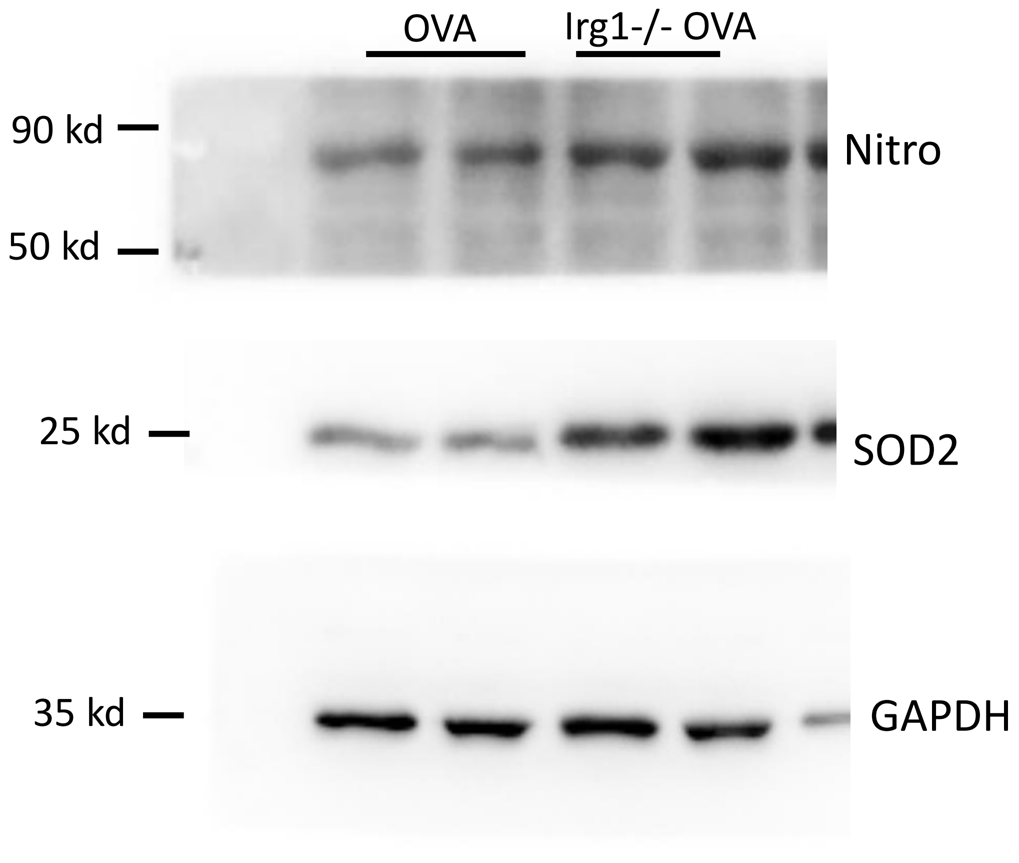

Figure S18. Raw blots of Figure 6H

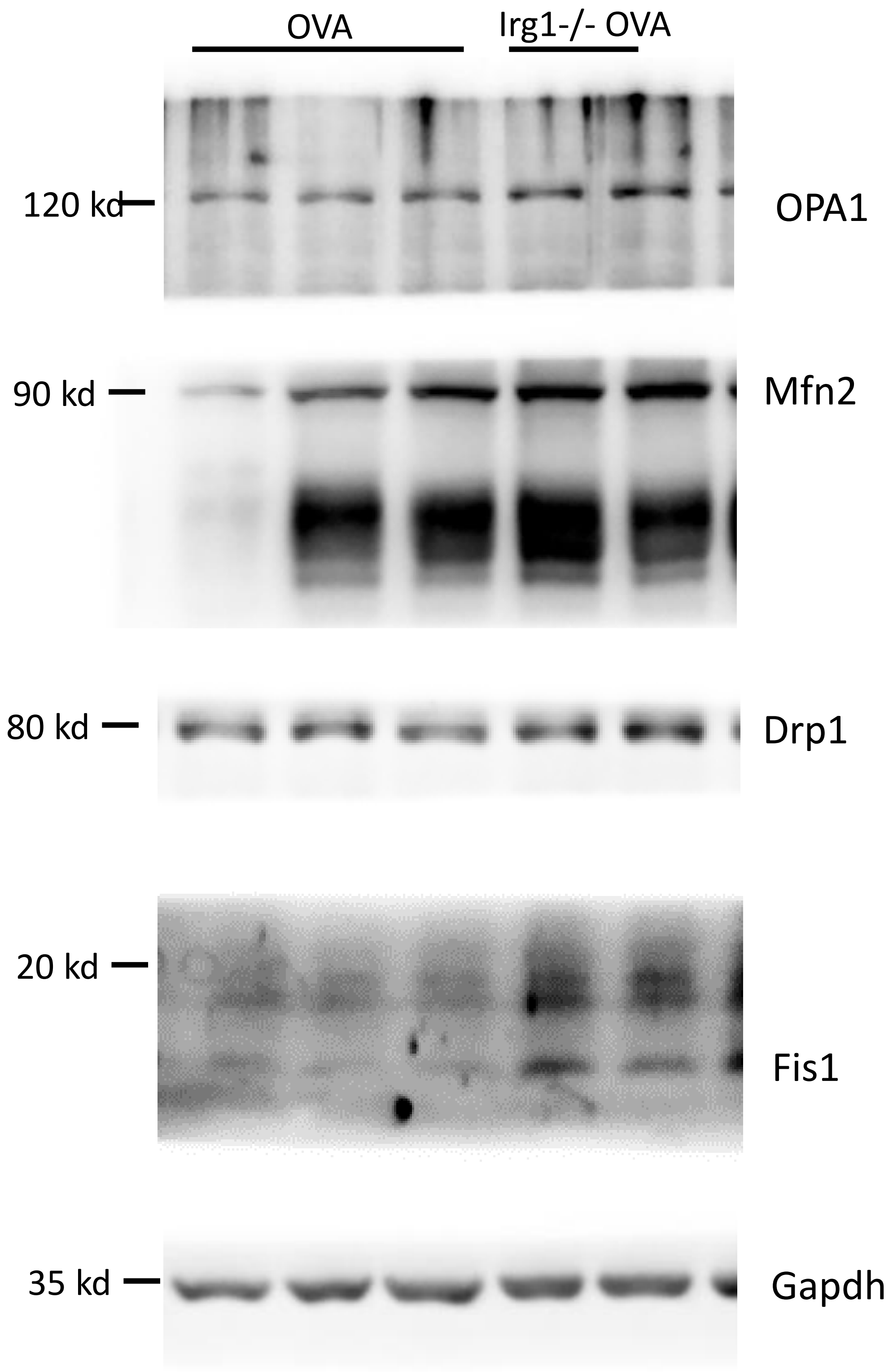

Figure S19. Raw blots of Figure 6I

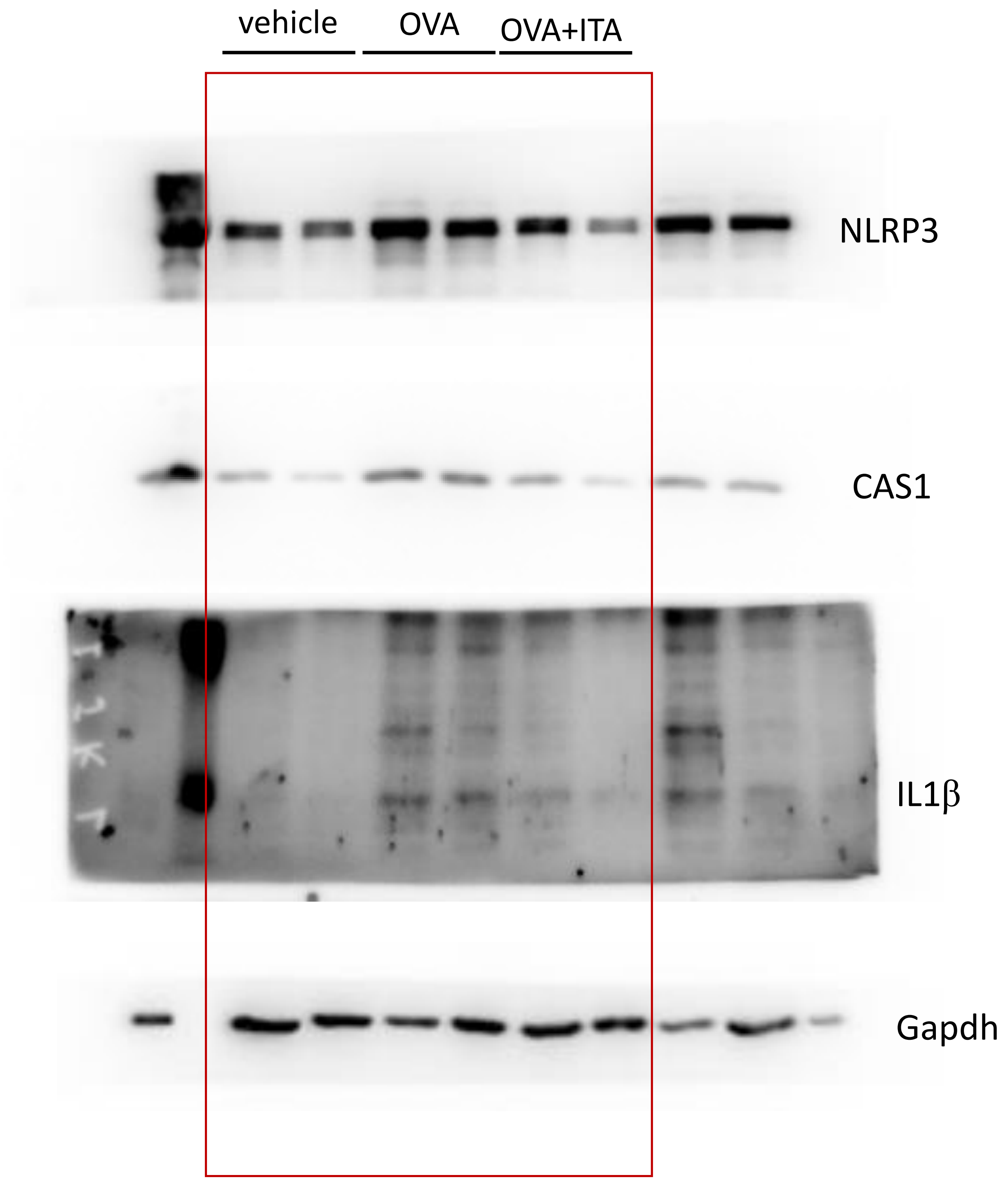

Figure S20. Additional bands used for statistics in Figure 6D

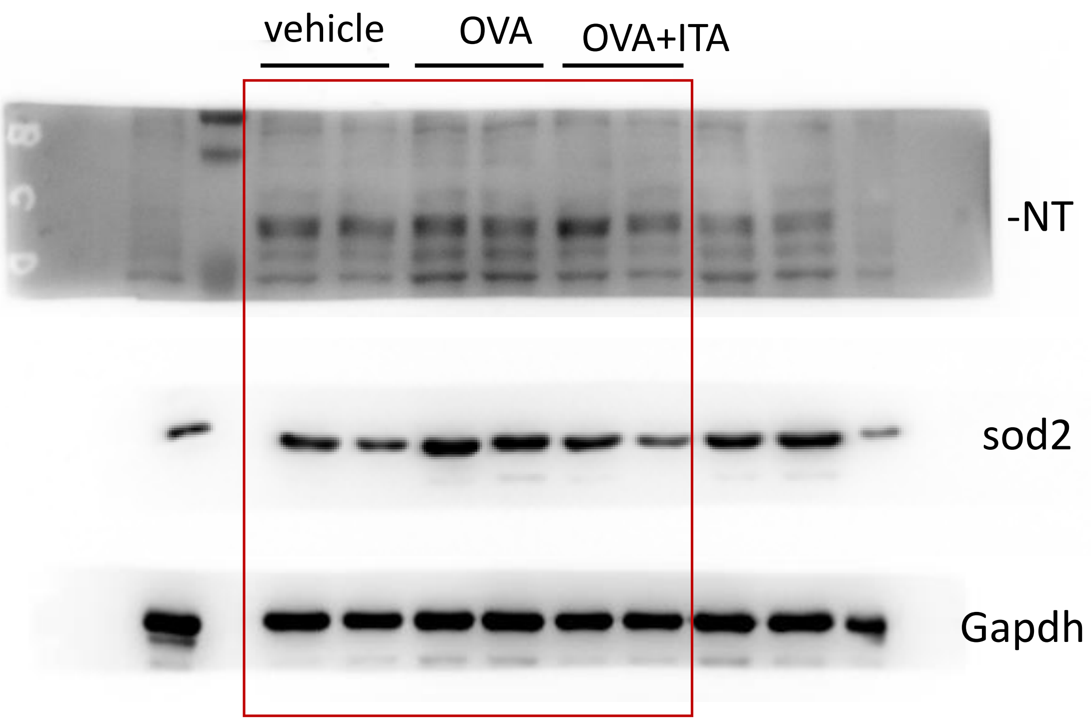

Figure S21. Additional bands used for statistics in Figure 6E

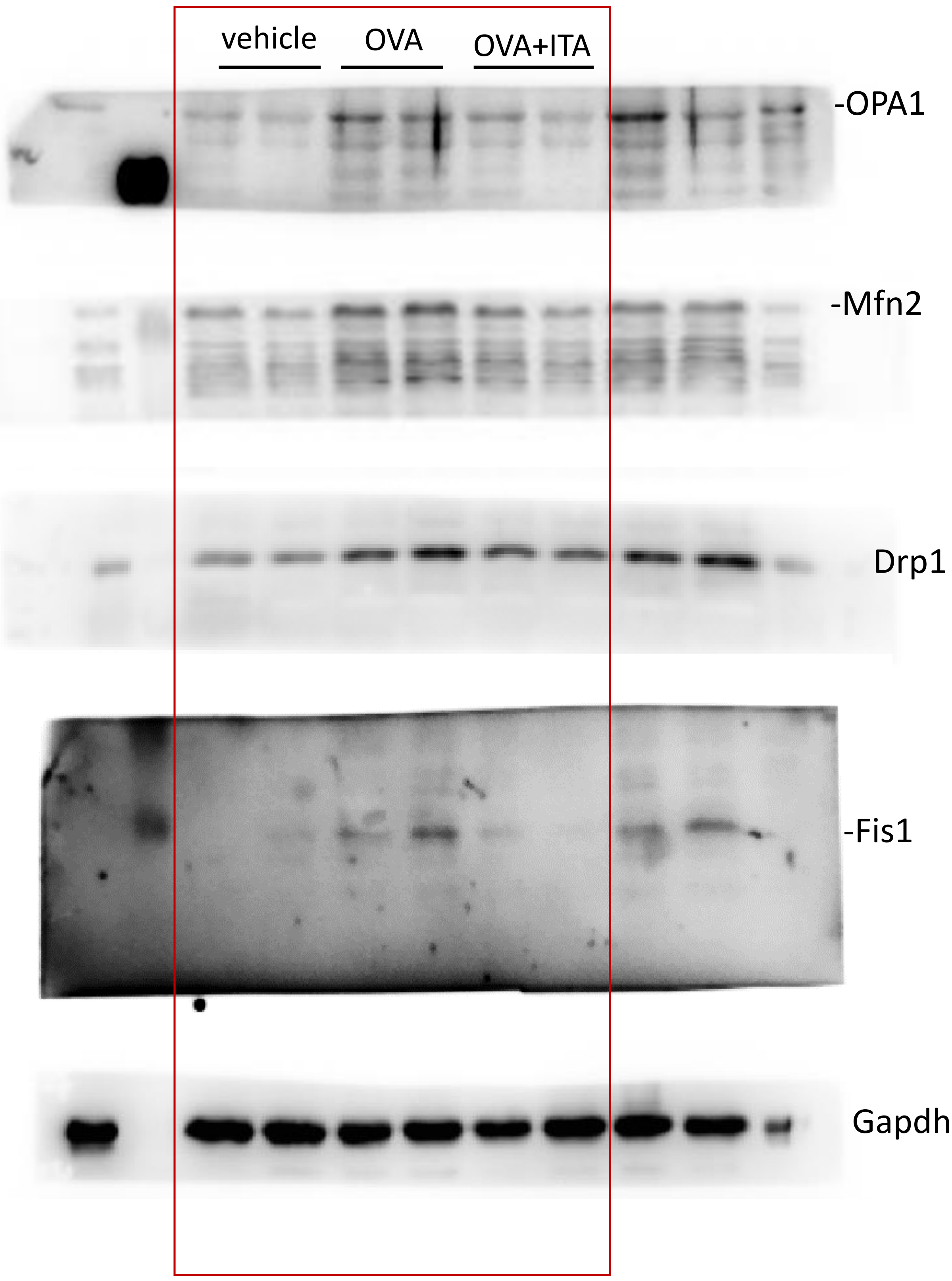

Figure S22. Additional bands used for statistics in Figure 6F

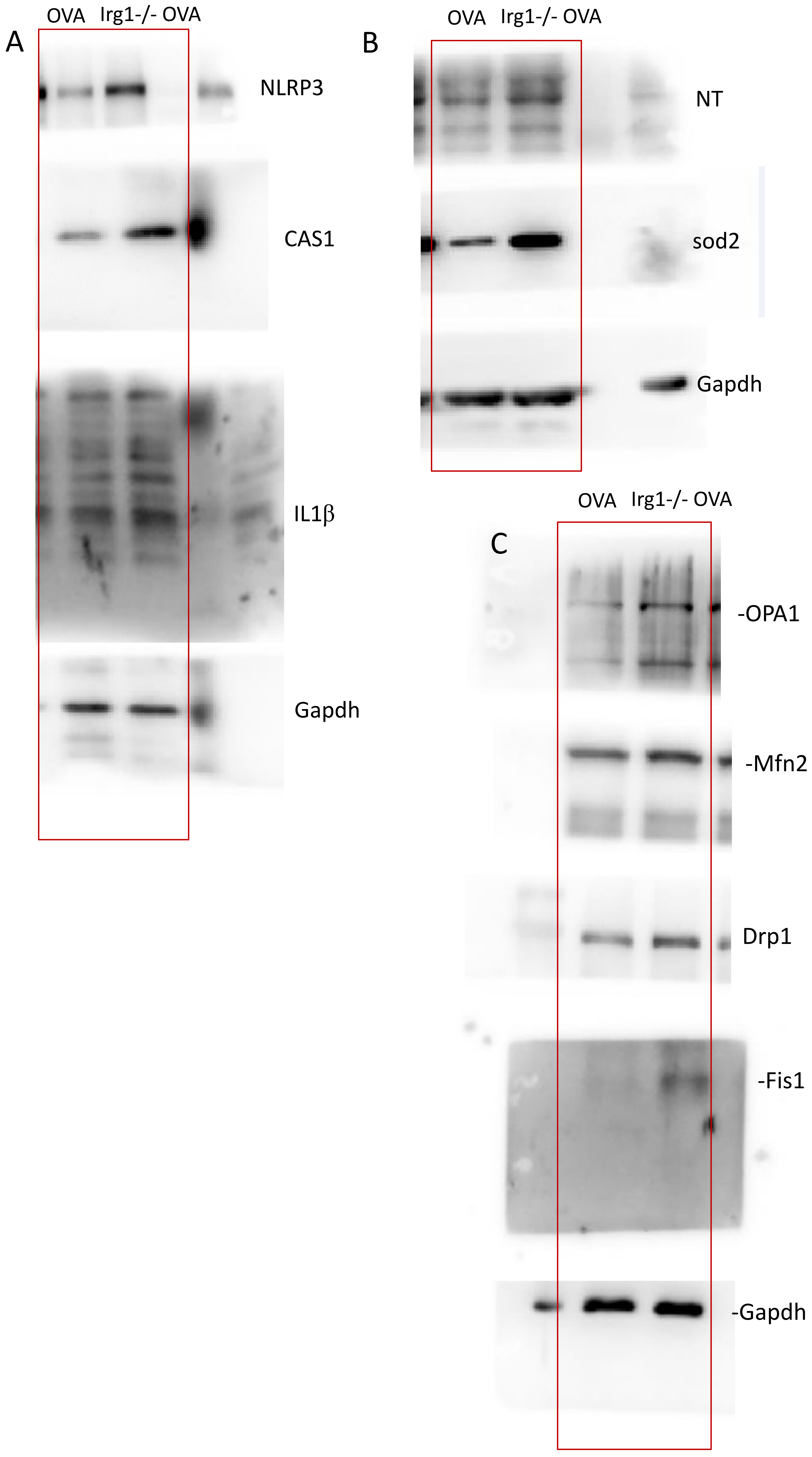

Figure S23. Additional bands used for statistics in Figure 6G, H, I

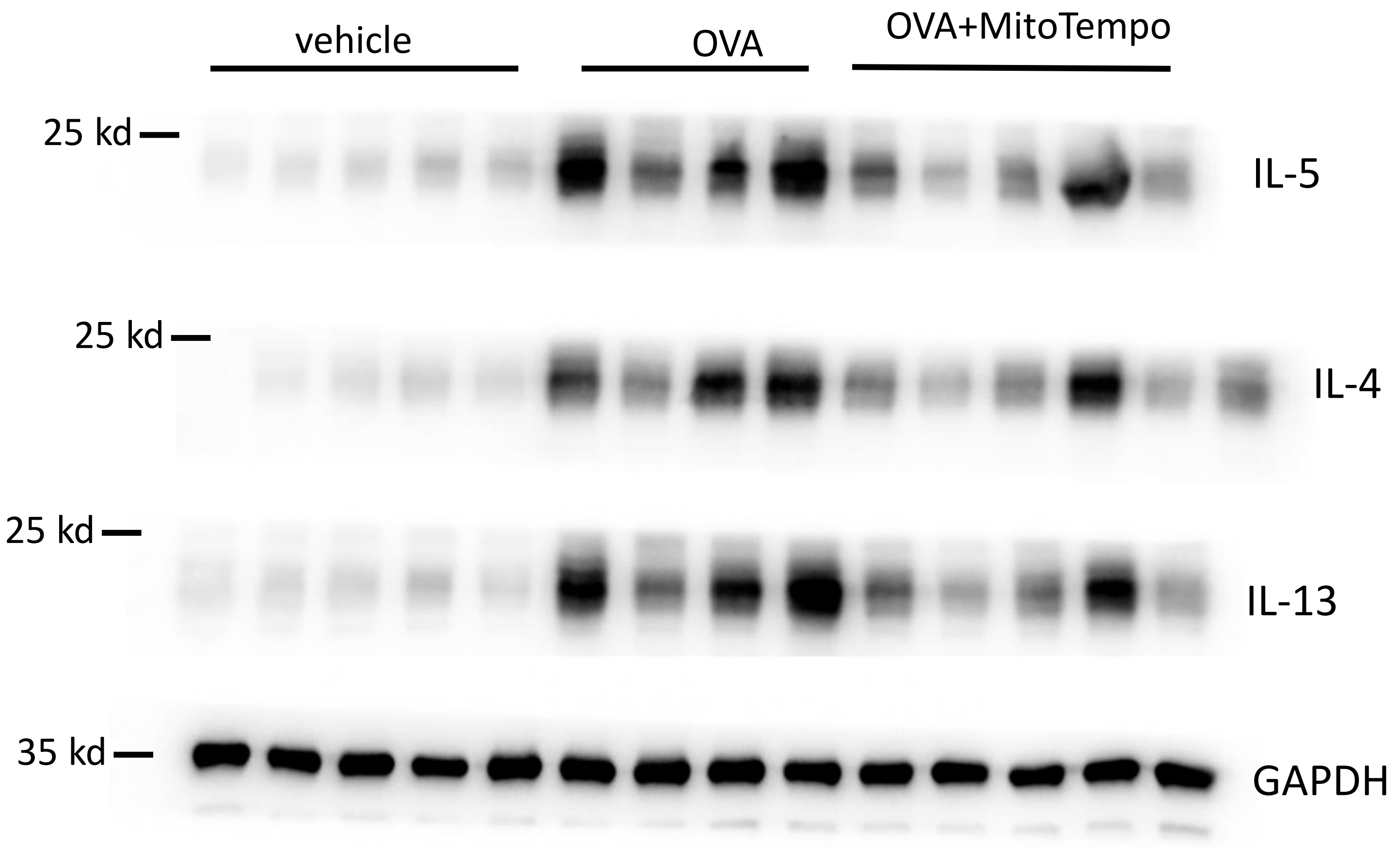

Figure S24. Raw blots of Figure S5 G

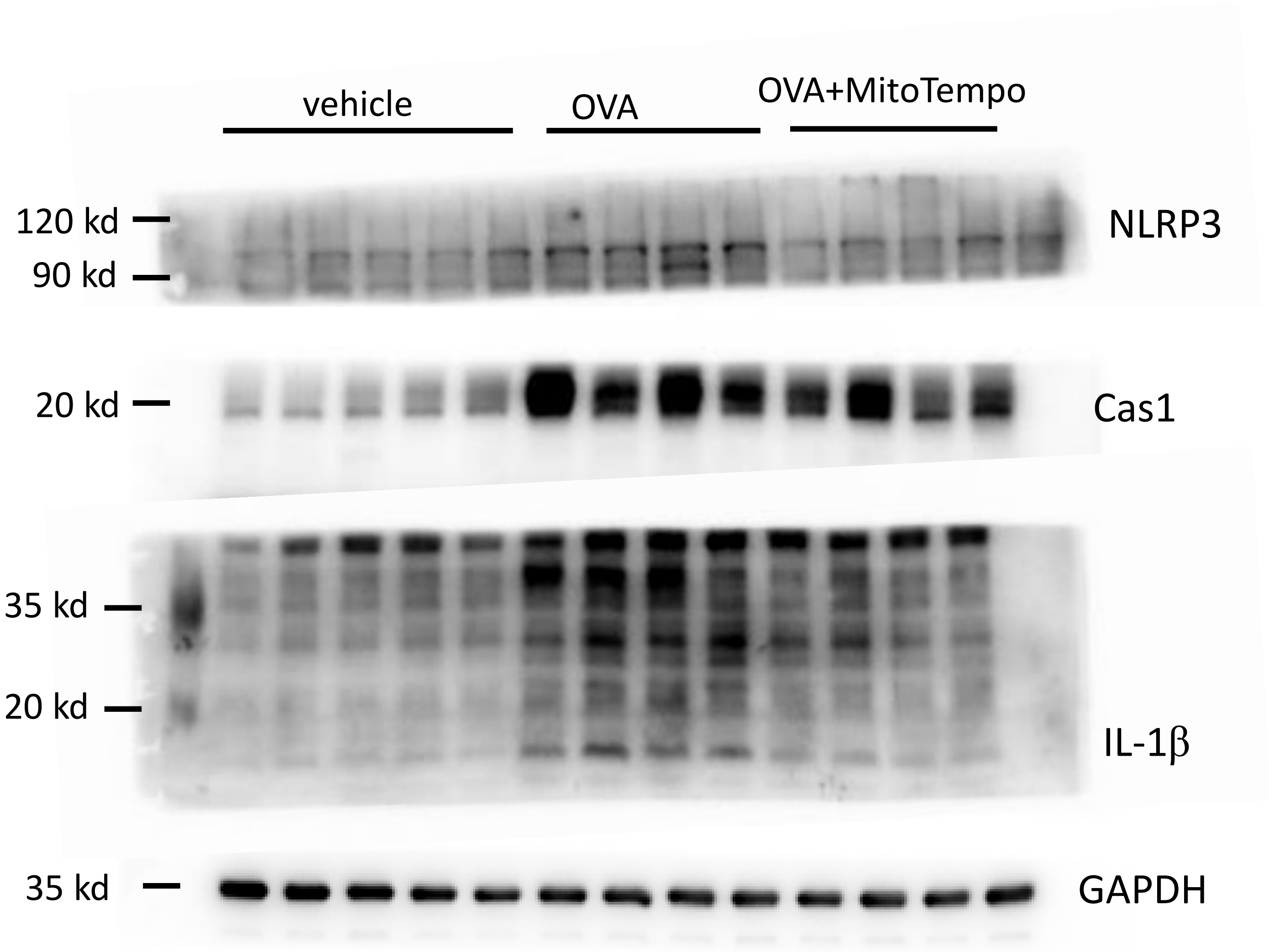

Figure S25. Raw blots of Figure S6 A

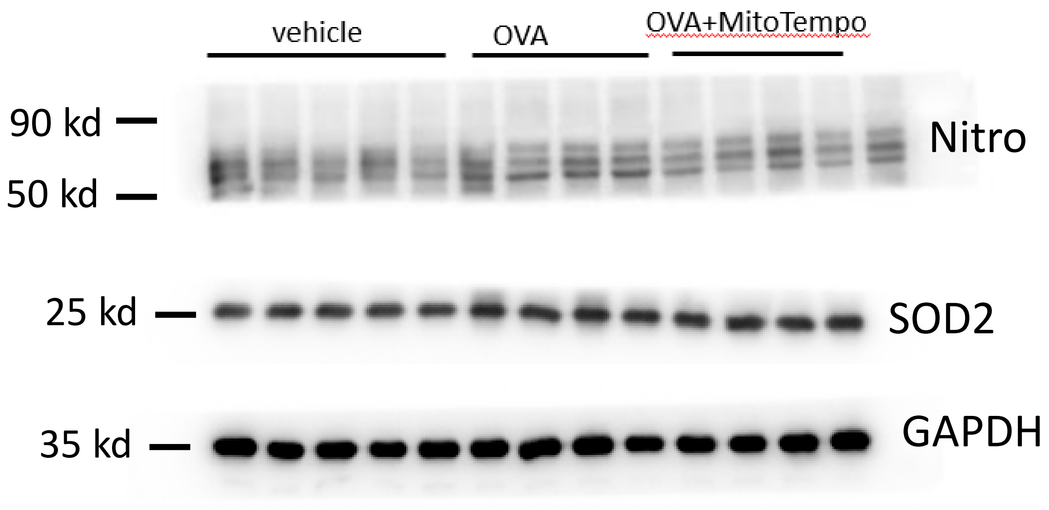

Figure S26. Raw blots of Figure S6 E

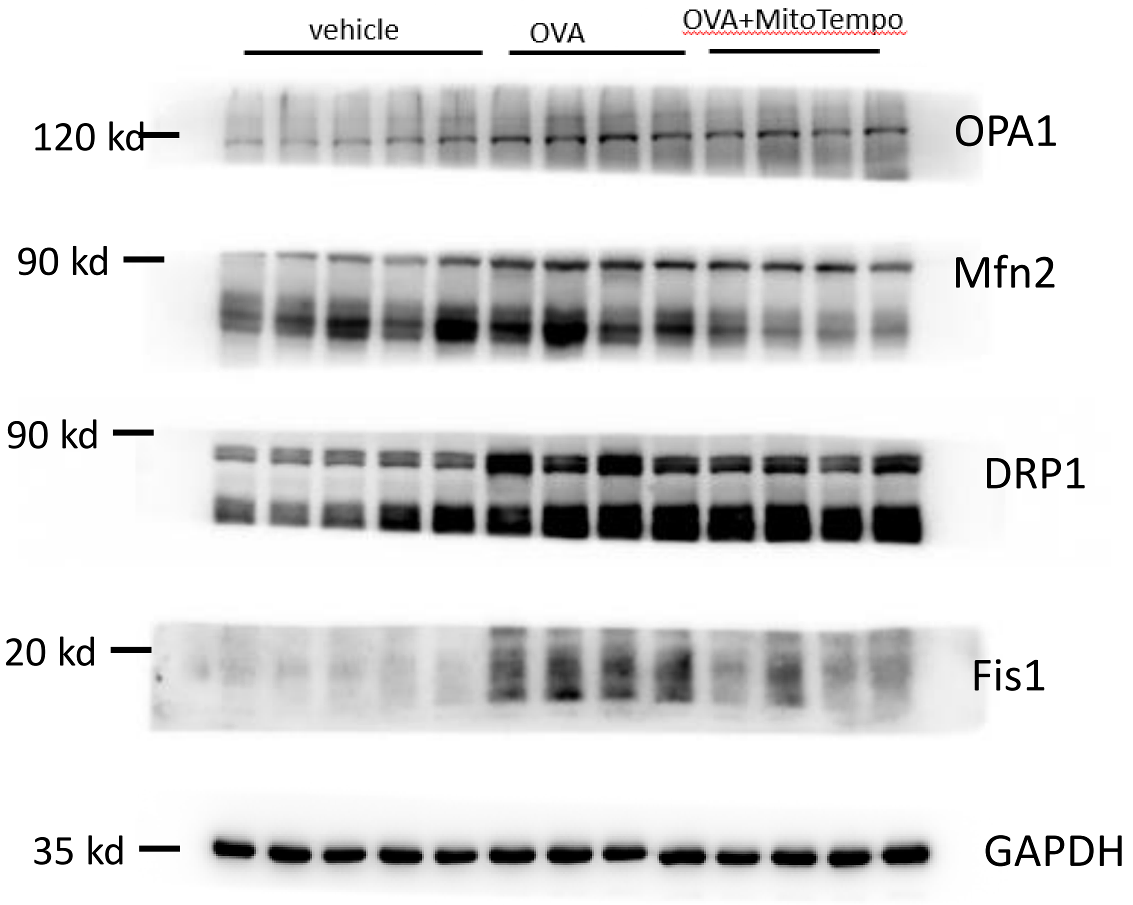

Figure S27. Raw blots of Figure S6 J
