# Supplementary material for: Itaconate Suppresses the Activation of Mitochondrial NLRP3 Inflammasome and Oxidative Stress in Allergic Airway Inflammation
Source: Antioxidants (Basel). 2023 Feb 15;12(2):489. doi: 10.3390/antiox12020489 (PMC9951851; doi:10.3390/antiox12020489)
Supplement: Supplementary file 1 [file antioxidants-12-00489-s001.zip › antioxidants-2129788-supplementary 3.pdf]

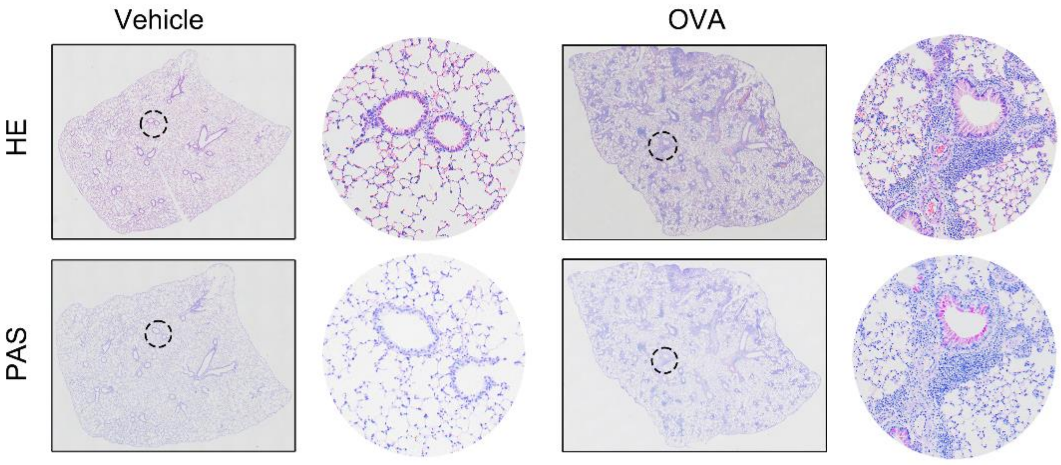

Figure S1. OVA-induced allergic airway inflammation. Representative whole lung sections stained with HE and PAS, scanned by ×10 objective.

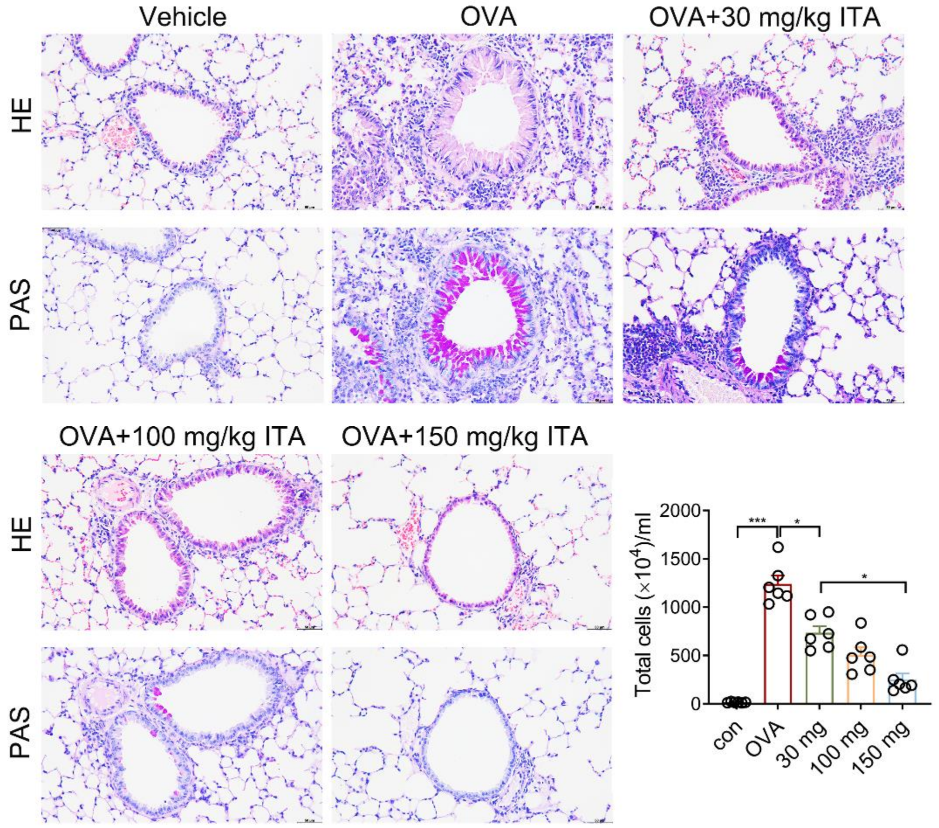

Figure S2. Itaconate (ITA) dose-depepently ameliorates OVA-induced allergic airway inflammation. Representative lung sections stained with HE and PAS, scale bar: 50 μm. And the number of total inflammatory cells in BALF. Data expressed as means ± SEM (n=6). *p<0.05, ***p<0.001.

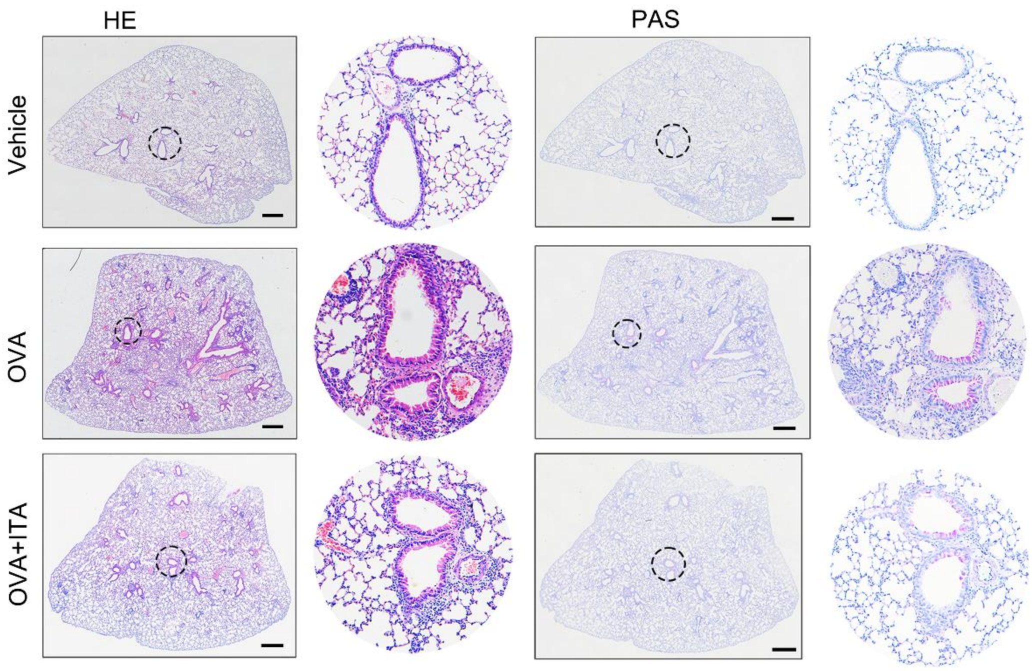

Figure S3. Representative whole lung sections stained with HE and PAS, scale bar: 500 μm.

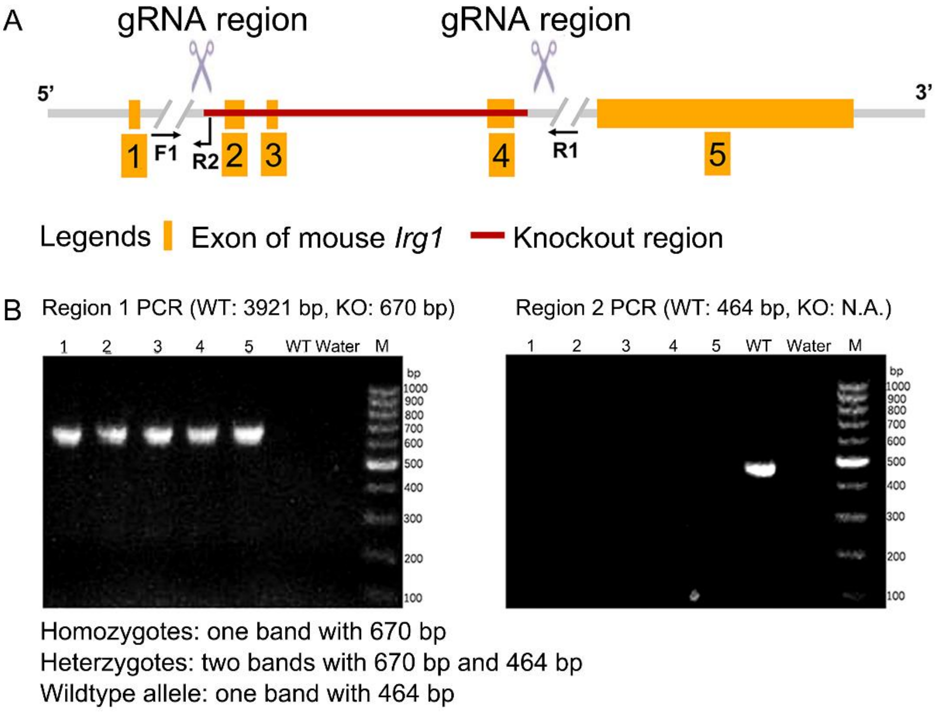

Figure S4. *Irg1*$^{-/-}$ mice available from Cyagen Biosciences Inc. (Stock# S-KO-02680, Suzhou, China). (A) Strategy for generation of *Irg1*$^{-/-}$ mice which deleted exons 2~4. (B) Homozygous *Irg1*$^{-/-}$ mice were generated by intercrossing the heterozygous mice and confirmed by PCR.

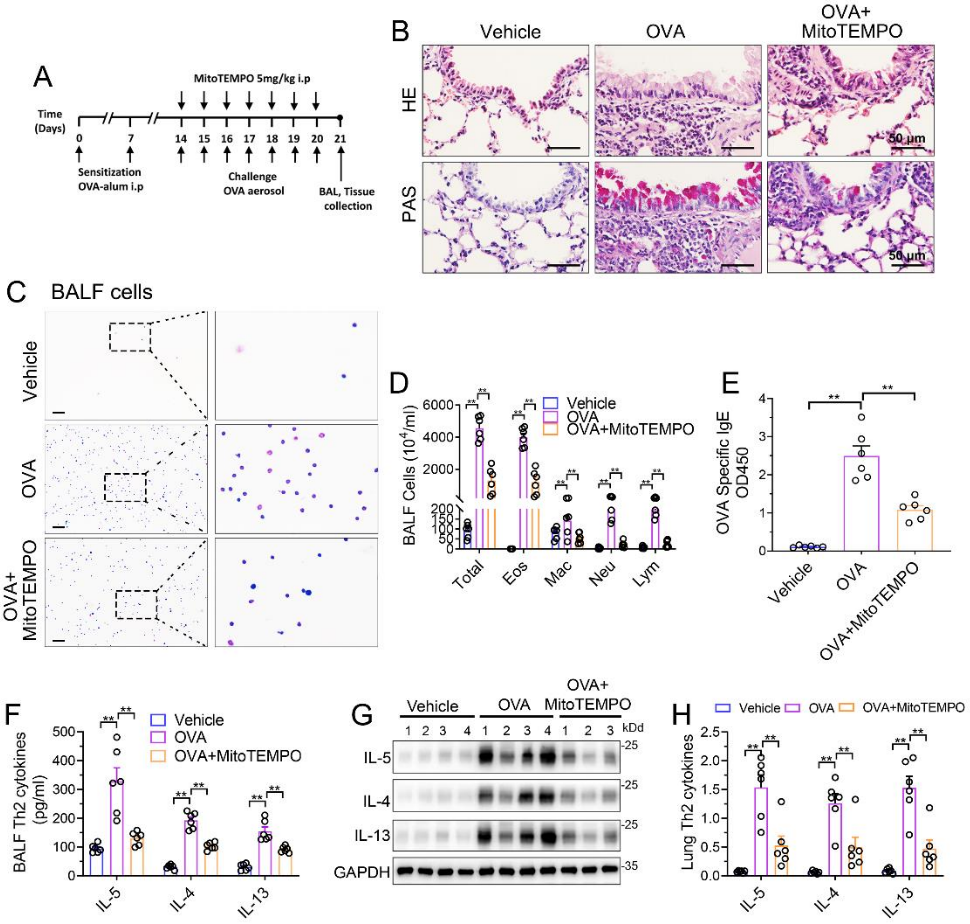

Figure S5. MitoTEMPO attenuates OVA-induced allergic airway inflammation. (A) Protocol for the establishment of OVA-induced allergic airway inflammation and treatment of MitoTEMPO. (B) Representative lung sections stained with HE and PAS, scale bar: 50 μm. (C) Representative images of BALF cells stained with Wright Gimesa solution. Scale bar: 100 μm. (D) The number of total inflammatory cells, eosinophils, macrophages, neutrophils and lymphocytes in BALF. (E) The level of IgE in serum. (F) The level of Th2 cytokines IL-5, IL-4 and IL-13 in BALF. (G, H) The protein level of Th2 cytokines IL-5, IL-4 and IL-13 in lung tissues. Cropped blots are shown, and Supplementary file 2 : Figure S24 presents the full-length blots. Data expressed as mean ±SEM (n=6). **p<0.01.

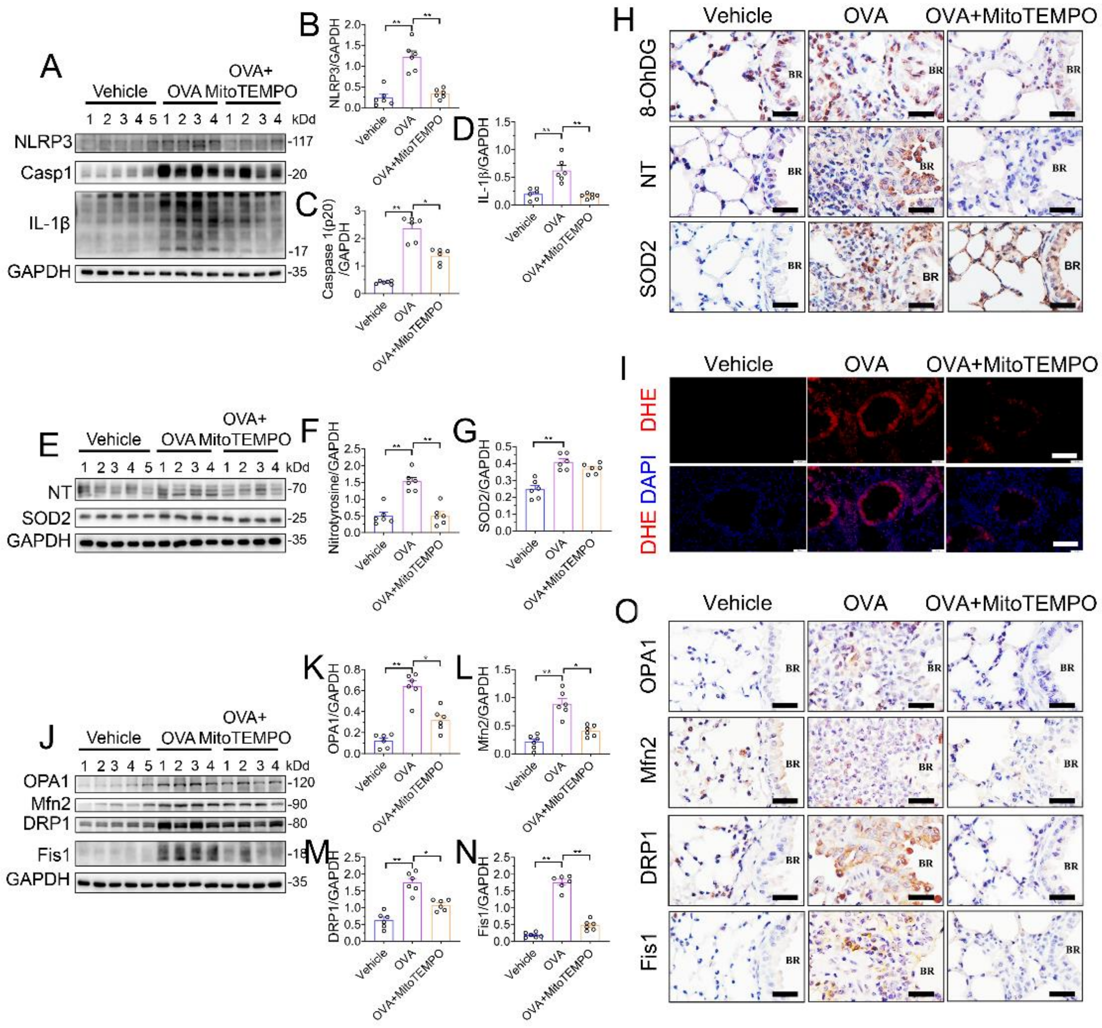

Figure S6. MitoTEMPO suppresses NLRP3 inflammasome activation, oxidative stress, mitochondrial dynamics in allergic airway inflammation. (A) Representative blots of NLPR3, Caspase-1(p20) and IL-1β(p17) in lung tissue. (B-D) Quantitative analysis of NLPR3, Caspase-1(p20) and IL-1β(p17) in lung tissue. (E) Representative blots of nitrotyrosine and SOD2 in lung tissue. (F, G) Quantitative analysis of nitrotyrosine and SOD2 in lung tissue. (H) Representative lung sections of the immunohistologic staining of 8-OHdG, nitrotyrosine and SOD2, scale bar: 50 µm. (I) Representative lung sections of DHE staining, Scale bar: 100 µm. (J) Representative blots of OPA1, Mfn2, DRP1 and Fis1 in lung tissue. (K-N) Quantitative analysis of OPA1, Mfn2, DRP1 and Fis1 in lung tissue. (O) Representative lung sections of the immunohistologic staining of OPA1, Mfn2, DRP1 and Fis1, scale bar: 50 µm. Cropped blots are shown, and Supplementary file 2 : Figure S25-27 presents the full-length blots. Data expressed as mean±SEM (n=6). *p<0.05, **p<0.01.
